# Tuning the Length of Filomicelles Prepared Via Templated Polymerization‐Induced Self‐Assembly

**DOI:** 10.1002/smll.74648

**Published:** 2026-07-17

**Authors:** Eleonora G. Hochreiner, Wiktoria Pioruńska, Gaëlle Mellot, Bence Fehér, Jean‐Michel Guigner, Viktoriia Drebezghova, Christophe Sinturel, Cornelus F. van Nostrum, Tina Vermonden, Jutta Rieger, Bas G. P. van Ravensteijn

**Affiliations:** ^1^ Division of Pharmaceutics, Utrecht Institute For Pharmaceutical Sciences, Faculty of Science Utrecht University Utrecht the Netherlands; ^2^ Institut Parisien de Chimie Moléculaire (IPCM) Centre national De La Recherche Scientifique (CNRS) Sorbonne Université Paris France; ^3^ Department of Chemical Engineering and Chemistry, Institute for Complex Molecular Systems (ICMS) Eindhoven University of Technology Eindhoven The Netherlands; ^4^ Institut De Minéralogie, De Physique des Matériaux et De Cosmochimie (IMPMC), Centre national De La Recherche Scientifique (CNRS) Sorbonne Université Paris France; ^5^ Interfaces, Confinement, Matériaux et Nanostructures (ICMN), Centre national De La Recherche Scientifique (CNRS) Université D'orléans Orléans France

**Keywords:** atomic force microscopy (AFM), block copolymer micelles, fluorescence (cross‐)correlation spectroscopy (F(C)CS), polymerization‐induced self‐assembly (PISA), supramolecular assembly, wormlike micelles

## Abstract

High‐aspect‐ratio polymeric micelles (filomicelles, FMs) are attractive anisotropic nanoparticles for a wide range of applications, however, their formation is typically confined to narrow hydrophilic/hydrophobic polymer block ratios, limiting systematic structure–property investigations. Consequently, independent control over micelle width, length, and stiffness remains challenging. A supramolecular polymerization‐induced self‐assembly (PISA) strategy using a bis‐urea core template was recently shown to robustly yield wormlike micelles across a wide range of core block lengths; however, the resulting FM lengths remained highly polydisperse. Herein, we investigate two strategies to tune the contour length: (***i***) Blending in polymer without the bis‐urea sticker and (***ii***) post‐micellization sonication. Unexpectedly, incorporating non‐sticker polymer can increase the contour length. Transmission electron microscopy (TEM), fluorescence cross‐correlation spectroscopy (FCCS), and high‐resolution atomic force microscopy (AFM) were used to elucidate the key factors contributing to this peculiar self‐assembly mechanism. Sonication‐induced cleavage led to shortening of the particles and narrower polydispersity in length. Combined, the two strategies provide access to a wide range of anisotropic micelles with accessible aspect ratios ranging from 1 to >150. Considering their long‐term (colloidal) stability, the presented system is an ideal model to study the effect of micellar anisotropy, for example, in drug delivery relevant scenarios.

## Graphical abstract

High‐aspect‐ratio polymeric micelles are attractive yet notoriously difficult to control in length. We reveal a counterintuitive and powerful solution: in supramolecularly templated PISA, adding non‐sticker polymer unexpectedly elongates the micelles, while post‐assembly sonication enables precise shortening with reduced dispersity. This dual strategy affords stable micelles with broadly tunable aspect ratios (1 to >150), enabling systematic studies of anisotropy‐mediated performance.

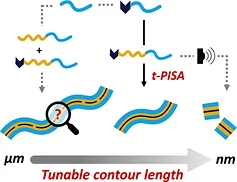

## 1 Introduction

Polymeric filamentous nanoparticles, or filomicelles (FMs), are elongated nanoparticles based on self‐assembled amphiphilic block copolymers (BCPs). This synthetic nanoparticle (NP) type mimics various biologically relevant nanostructures such as filoviruses [1], and has shown different behavior in flow and cellular uptake pathways compared to their spherical counterparts [2, 3]. FMs have been studied as nanocarriers for drug delivery due to their high drug loading capacities and morphology‐dependent circulation times in vivo [4, 5, 6]. However, translation toward therapeutic or even clinical applications has been limited by challenges in the robust and scalable production of FMs, as well as batch‐to‐batch variability [6].

Typically, the preparation of FMs requires the synthesis of BCPs with specific volumetric aspect ratios between the hydrophilic and the hydrophobic polymer blocks. This ratio is defined by the packing parameter *p = v/(a_0_l)*, where *a_0_* is the optimal interfacial area occupied by the hydrophilic block and *l* and *v* are the length and volume, respectively, of the hydrophobic segment. In the case of ideal chain packing, ⅓ < *p* < ½ is expected to result in wormlike micelles [7]. The predictive power of these geometric arguments is limited due to unknown scaling relations linking block segment lengths to packing parameters, the disregard of entropic contributions, and overruling of thermodynamic equilibrium by kinetic trapping [8, 9]. Finding the typically narrow experimental window in which wormlike micelles are formed requires tedious optimization of the self‐assembly conditions. In this respect, polymerization‐induced self‐assembly (PISA), where BCP synthesis and self‐assembly into particles occur simultaneously, offers an attractive alternative to the conventional solvent‐exchange or thin‐film rehydration methods [10, 11, 12, 13, 14]. Growth of the core‐forming block causes a gradual change of the amphiphilic balance of the BCPs. This method drives morphological transitions of the self‐assembled structures, typically showing an evolution from spherical particle to polymer vesicles with an intermediate worm‐rich phase [15]. Although PISA enables rapid screening of block ratios and high–solid‐content dispersions [16, 17], it does not widen the experimental window in which FMs form. As a result, FMs typically coexist with (intermediate) morphologies and exhibit only limited tunability in thickness, contour length, and flexibility.

Chemical strategies to overcome packing‐parameter constraints and broaden the experimental window for worm formation in PISA have largely focused on two approaches. These rely either on crystallinity, as in polymerization‐induced crystallization‐driven self‐assembly (PI‐CDSA) [18, 19, 20], or on introducing anisotropic secondary interactions into the solvophobic polymer segment, such as hydrogen bonding [21, 22], electrostatic [23], or π–π interactions [24, 25, 26, 27]. Preventing morphological transitions to vesicles by either in situ core‐crosslinking [28, 29] or kinetically controlling the PISA process [30] also falls within the realm of possibilities.

Mellot et al. introduced an efficient approach by exploiting supramolecular hydrogen bonding to produce wormlike micelles/fibers via templated PISA (*t*‐PISA). Rather than relying on additional secondary interactions in the entire hydrophobic repeat unit, a bis‐urea sticker was introduced at the BCP end in the core of the particles. Acting as a template during self‐assembly, it imprints directionality and steers the assembly process to favor a cylindrical morphology [31, 32]. The BCP (poly(*N,N*‐dimethylacrylamide)‐*block*‐poly(2‐methoxyethyl acrylate) (p(DMAc)‐*b*‐p(MEA)) was prepared via aqueous reversible addition‐fragmentation chain transfer (RAFT)‐PISA using p(DMAc)‐based macro‐chain transfer agents (macroCTAs) that carried the sticker moiety. Pure worm phases were obtained for a wide variety of block ratios and monomer concentrations. For example, variation of the length of the core forming block (degree of MEA polymerization, *DP*_MEA_ ≈ 50–220) led to tunability of the fiber diameter (*d* = 16–28 nm). Additionally, employing poly(acrylic acid) (p(AA)) instead of p(DMAc) as the hydrophilic, stabilizing block still resulted in long fibers, hinting at the versatility of this approach. However, regardless of the choice of BCP composition or reaction conditions, the contour length remained uncontrolled and largely polydisperse.

In this work, we demonstrate tunability of the contour length of these p(DMAc)‐*b*‐p(MEA)‐based FMs by exploring two strategies. (***i***) Synthesizing fibers using a mixture of CTAs with and without the sticker moiety. This route was inspired by the use of end‐cappers in one‐dimensional supramolecular assembly processes [33, 34, 35]. The second strategy (***ii***) entails sonication‐induced worm scission post‐*t*‐PISA. By systematically analyzing the *t*‐PISA process using a combination of (cryo‐)transmission electron microscopy (TEM), fluorescence cross‐correlation spectroscopy (FCCS), and high‐resolution atomic force microscopy (AFM), and by evaluating the robustness and synthetic scope of these strategies, we aimed to generate a library of chemically (near‐)identical FMs with widely tunable aspect ratio. Such length‐controlled libraries make it possible to unambiguously probe how particle anisotropy dictates performance in applications where shape is critical, a capability that was previously unattainable.

## 2 Results and Discussion

### 2.1 Templated‐PISA: Sticker‐Guided Micellar Morphology

**FIGURE 1:**
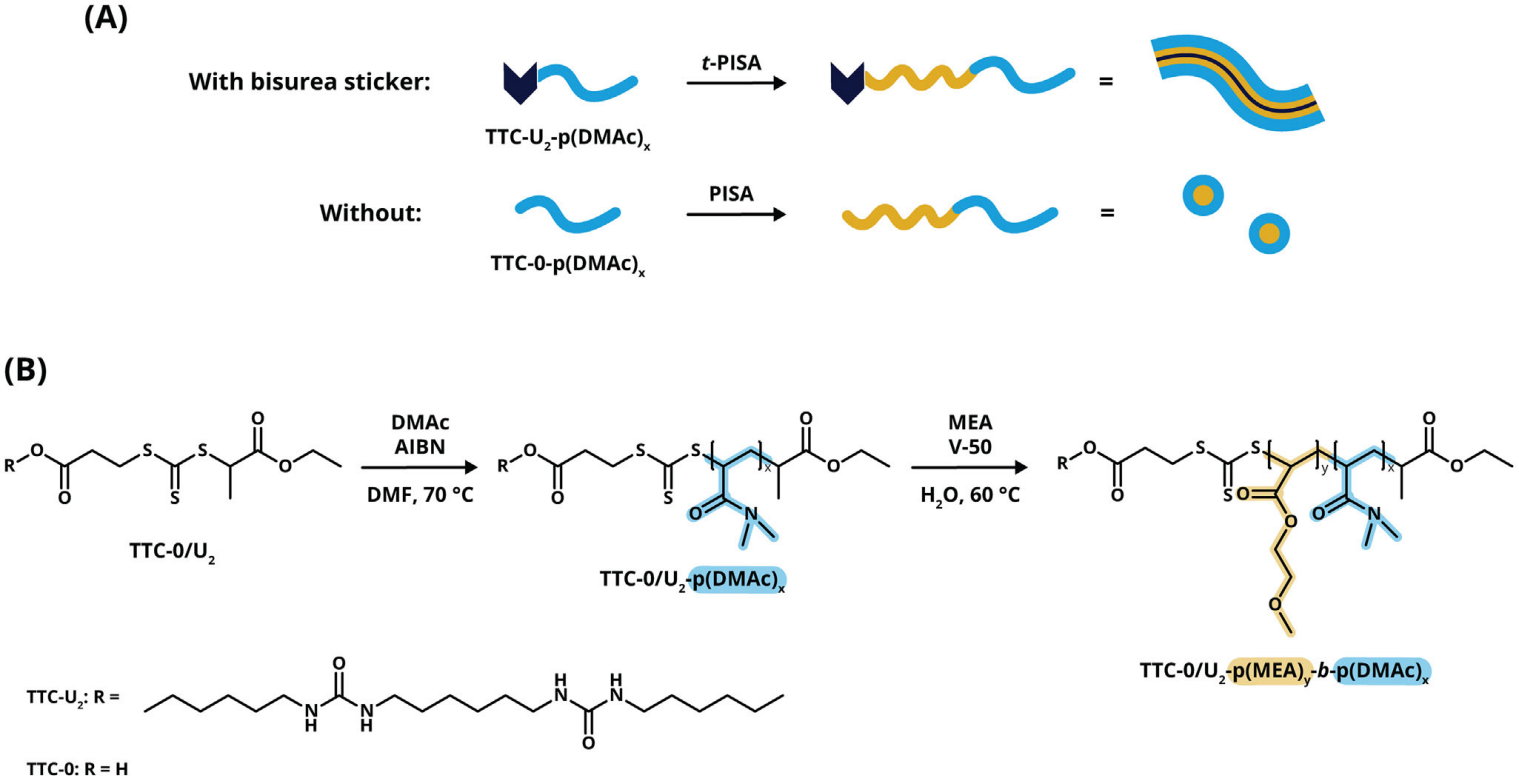
(A) Schematic representation of (templated) PISA and obtained particles depending on the presence of the bis‐urea sticker. (B) Synthesis of TTC‐p(MEA)_y_‐*b*‐_p_(DMAc)_x_ starting from the CTAs TTC‐0 or TTC‐U_2_, via the preparation of the corresponding macroCTAs TTC‐0‐p(DMAc)_x_ or TTC‐U_2_‐p(DMAc)_x_.

First, two macromolecular chain transfer agents (macroCTAs) based on poly(*N*,*N*‐dimethyl acrylamide) (pDMAc) were synthesized starting from TTC‐U_2_ and TTC‐0; two CTAs with and without a bis‐urea sticker, respectively (Figure 1 and S1–S3; TTC refers to the trithiocarbonate‐type of CTA), following the procedure described by Mellot et al. [31]. Shortly, DMAc was polymerized via RAFT polymerization in *N*,*N*‐dimethylformamide (DMF) at 70°C with 2,2′‐azobis(isobutyronitrile) (AIBN) as radical initiator in the presence of either TTC‐0 or TTC‐U_2_. After 75 min of reaction, monomer conversions of 77% for TTC‐U_2_‐p(DMAc)_33_ and 69% for TTC‐0‐p(DMAc)_29_ were obtained by proton nuclear magnetic resonance (^1^H‐NMR) spectroscopy, which corresponds to number‐average molar mass (*M*_n, th_) of 3.9 and 3.2 kg/mol, respectively (Table S1). The products were purified via dialysis against water, followed by isolation with freeze‐drying and characterization by ^1^H‐NMR and size exclusion chromatography (SEC) (Figure S3 and S7).

Both macroCTAs were separately used in a subsequent PISA process to verify their equal reactivity and ability to control a RAFT polymerization (**P1** and **P6**, Table 1). To this end, chain extension reactions with 2‐methoxyethyl acrylate (MEA) targeting a degree of polymerization (DP) of 99 were performed at 60°C with 2,2′‐azobis(2‐methylpropionamidine) dihydrochloride (V‐50) as radical initiator. ^1^H‐NMR confirmed MEA conversions exceeding 90% after 140 min for both macroCTAs (Table 1, entries **P1** and **P6**). Monitoring the MEA conversion as a function of the reaction time revealed comparable polymerization kinetics (Figure S5, S6). Furthermore, SEC analysis confirmed good chain extension to BCPs for both sticker and non‐sticker containing macroCTAs (Figure S7). Together these results indicate that the sticker end‐group has no significant influence on the effectiveness of the CTA, allowing for an artifact‐free study of the mixing ratio of sticker: non‐sticker CTA on the contour length of the resulting micelles as will be discussed below.

**TABLE 1:** Overview of the aqueous templated‐PISA (**P**) reactions performed to generate the anisotropic particle library.

Sample^a^	TTC‐0: TTC‐U_2_ ^b^	*X*_MEA_ ^c^ (%)	*DP*_MEA_, _NMR_ ^d^ (‐)	*M*_n, th_ ^d^ (kg/mol)	*M*_n, SEC_ ^e^ (kg/mol)	*M*_w, SEC_ ^e^ (kg/mol)	*Đ* ^e^ (‐)	Morphology^f^
**P1**	0:100	95	94	16.4	9.9	12.0	1.22	Fibers
**P2**	50:50	84	83	14.0	9.5	11.8	1.24	Fibers
**P3**	70:30	96	95	15.8	9.0	11.9	1.33	Fibers
**P4**	80:20	95	94	15.6	8.9	12.1	1.35	Fibers
**P5**	90:10	97	96	15.7	8.2	10.8	1.31	Fibers
b	100:0	93	92	15.1	9.2	11.0	1.20	Spheres

^a^ [MEA]_0_/[TTC‐p(DMAc)]_0_ = 99, [MEA]_0, aq_ = 0.86 M, [TTC]_0_/[AIBN]_0_ = 10.

^b^ The molar ratio between TTC‐0 and TTC‐U_2_ were calculated based on the *M*_n, th_ of the individual macroCTAs.

^c^ MEA conversion (*X*_MEA_) was determined at the end of the reaction (140 min) by ^1^H‐NMR, using 1, 3, 5‐trioxane as internal standard.

^d^ Theoretical degree of polymerization (*DP*) and number‐average molar mass, *M*_n_, based on the experimentally determined MEA conversion.

^e^ Number‐average molar mass, *M*_n_, weight‐average molar mass, *M*_w_, and dispersity index, *Đ*, determined by SEC in DMF (+ 10 mM LiCl) using PEG standards for calibration.

^f^ Determined by TEM of aqueous NP dispersions (1 mg/mL) after staining with 2% aqueous uranyl acetate solution.

**FIGURE 2:**
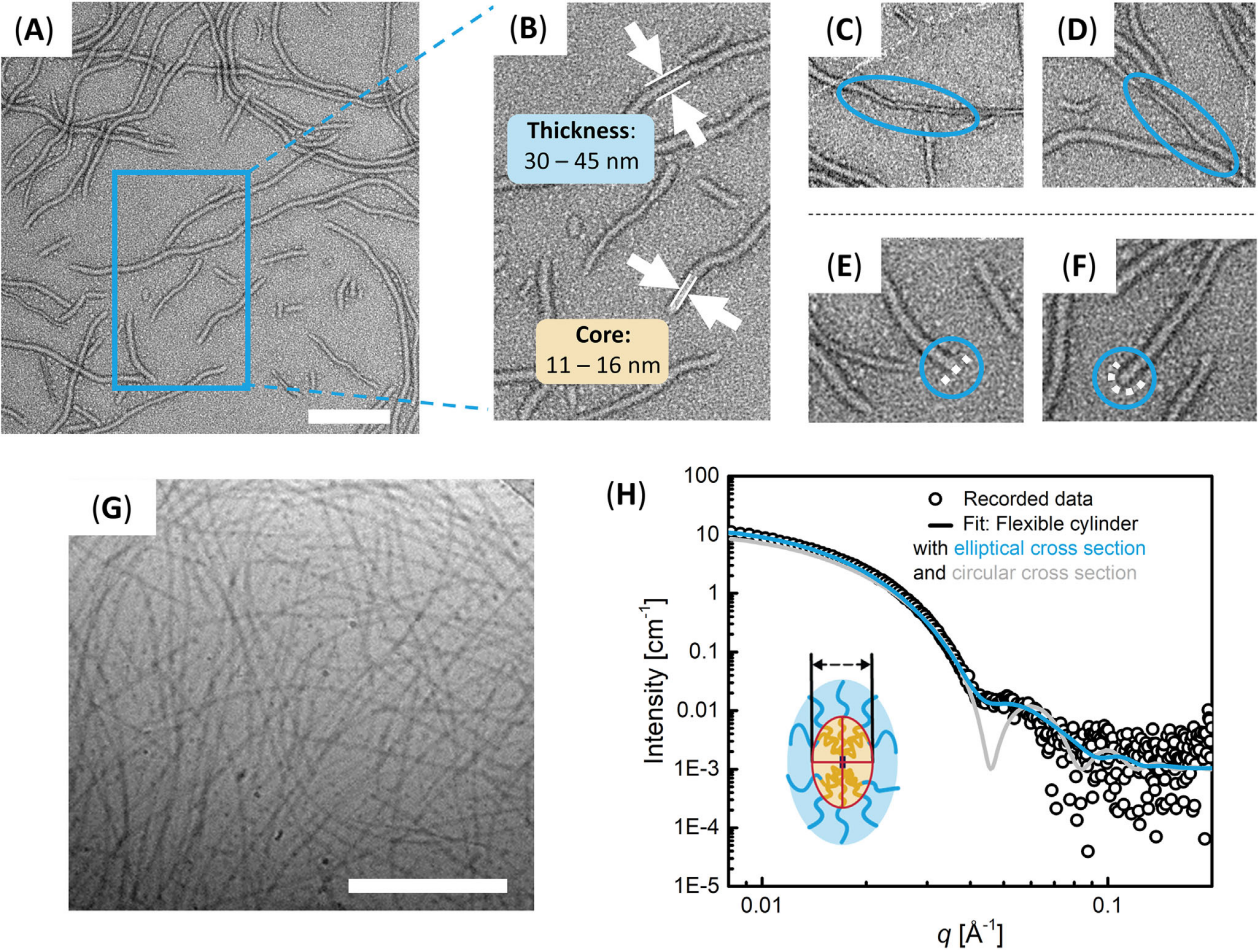
(A) Dry‐state TEM micrograph of TTC‐U_2_‐p(MEA)_94_‐*b*‐p(DMAc)_33_ (**P1**). Scale bar = 500 nm. (B) Zoom‐in of the image shown in (A) to estimate the total and core thickness of the FMs. A distinction was made between the shell (dark, stained with uranyl acetate) and the core (lighter, no stain). High magnification images to show (C, D) coexistence of thinner segments along the contour length as evidence for the core ellipticity and (E, F) examples of fibers with open and closed chain ends. (G) Cryo‐TEM micrograph of TTC‐U_2_‐p(MEA)_94_‐*b*‐p(DMAc)_33_ (**P1**). Scale bar = 500 nm. (H) SAXS data (open symbols) of **P1** at RT. Data were fitted with a model for a flexible cylinder with an elliptical cross section (blue curve) and a circular cross section (gray curve).

Analyzing the morphology of the micellar structures with TEM revealed the anticipated spherical and wormlike morphology for the non‐sticker‐ (Table 1, entry **P6**, Figure S10) and sticker‐containing (BCPs) (Table 1, entry **P1**, and Figure 2A, Figure S9‐11), respectively [36]. As previously reported by Mellot et al. [31, 32], these results underline the requirement for the sticker to force the micellar growth in a pseudo‐1D fashion; without the sticker and near‐equal block ratios, the amphiphilic polymers assemble into typical spherical micelles. The formation of the wormlike morphology proved to be robust and reproducible (Figure S11).

To validate that these morphologies determined by dry‐state TEM are representative for the micellar state in aqueous solution, dynamic light scattering (DLS) was performed for the spherical micelles (**P6**) as there is no interference from particle anisotropy. Hydrodynamic radii (*R*_h_) of 14 nm with a low polydispersity index (PDI = 0.06) were obtained, in agreement with TEM (see Figure S10). For the FMs, cryo‐TEM and small angle x‐ray scattering (SAXS) analyses rather than DLS were performed as representative solution state techniques (Figures 2G and H) to accurately capture their anisotropic characteristics. For both morphologies, DLS, or cryo‐TEM, and SAXS data were in agreement with the appearance in dry‐state TEM, pointing towards the absence of drying and/or staining‐induced artifacts.

For the FMs, SAXS provided further structural characterization by fitting the scattering profile to that of a flexible cylinder (Figure 2H, gray and blue curves). Only scattering of the hydrophobic core was taken into account due to the negligible scattering contrast of the p(DMAc) outer corona with the aqueous dispersing medium. Unexpectedly, the profiles could be fitted more accurately by taking ellipticity of the cross section into account, where the long axis of the cross section is 1.4–1.5 times larger compared to the shorter equatorial radius (14–15 nm). Accurate measurements for the FM's lengths could not be extracted from the SAXS profiles due to a limitation of the lowest achievable *q*‐values and high length dispersity. We hypothesize that the core ellipticity optimizes the stacking efficiency of the bis‐urea sticker moieties sitting in the core. Close inspection of the dry‐state TEM pictures provided comparable estimates for the thicknesses (Figure 2B) and confirmed the elliptical cross‐section. Although the majority of the particles seem to lay down on the longer axis, most probably as a result of the drying during sample preparation, on multiple occasions thinner worm sections are observed along the contour length (Figures 2C,D). We attribute this to twisting of the FM, thereby exposing the shorter equatorial radius.

Given that dry‐state TEM confirmed the SAXS data in representing the particle morphology and its relative simplicity and high contrast, we relied on this technique coupled to manual tracing of the FMs (see Supporting Information Section 2.4) throughout the rest of the study to quantitatively measure contour lengths. The results are reported as the number‐based and weight‐based average lengths, *L*_n_ and *L*_w_, respectively, as well as *L*_w_/*L*_n_ as a measure of polydispersity. For **P1**, an average length (*L*_n_) of 305 ± 338 nm was found. The tracing procedure was found to be highly reproducible (see Figure S9).

### 2.2 End‐Capper Strategy: MacroCTA Combinations to Tune the Contour Length

TEM analysis revealed that in the **P1** sample, a fraction of the FMs seem to have cutoff or open ends (Figure 2E and Figure S8 for schematic representation), suggesting that these ends expose the hydrophobic core to the aqueous environment, which is energetically unfavorable. We hypothesize that these open ends are a direct consequence of the 1D stacking of the bis‐urea stickers in the core, which counteracts any bending of the BCPs within the FM. On the contrary, the polymers without the stickers have a natural tendency for curvature upon self‐assembly as they pack into spherical micelles with hydrodynamic diameters comparable to the FM thickness (see Figure S10). Inspired by end‐cappers used in supramolecular polymerizations [33, 34, 35], we speculated that the average contour length of the FMs could be controlled by varying the ratio of sticker to non‐sticker containing BCPs, where the non‐sticker polymers would disrupt 1D growth and thus limit the contour length.

**FIGURE 3:**
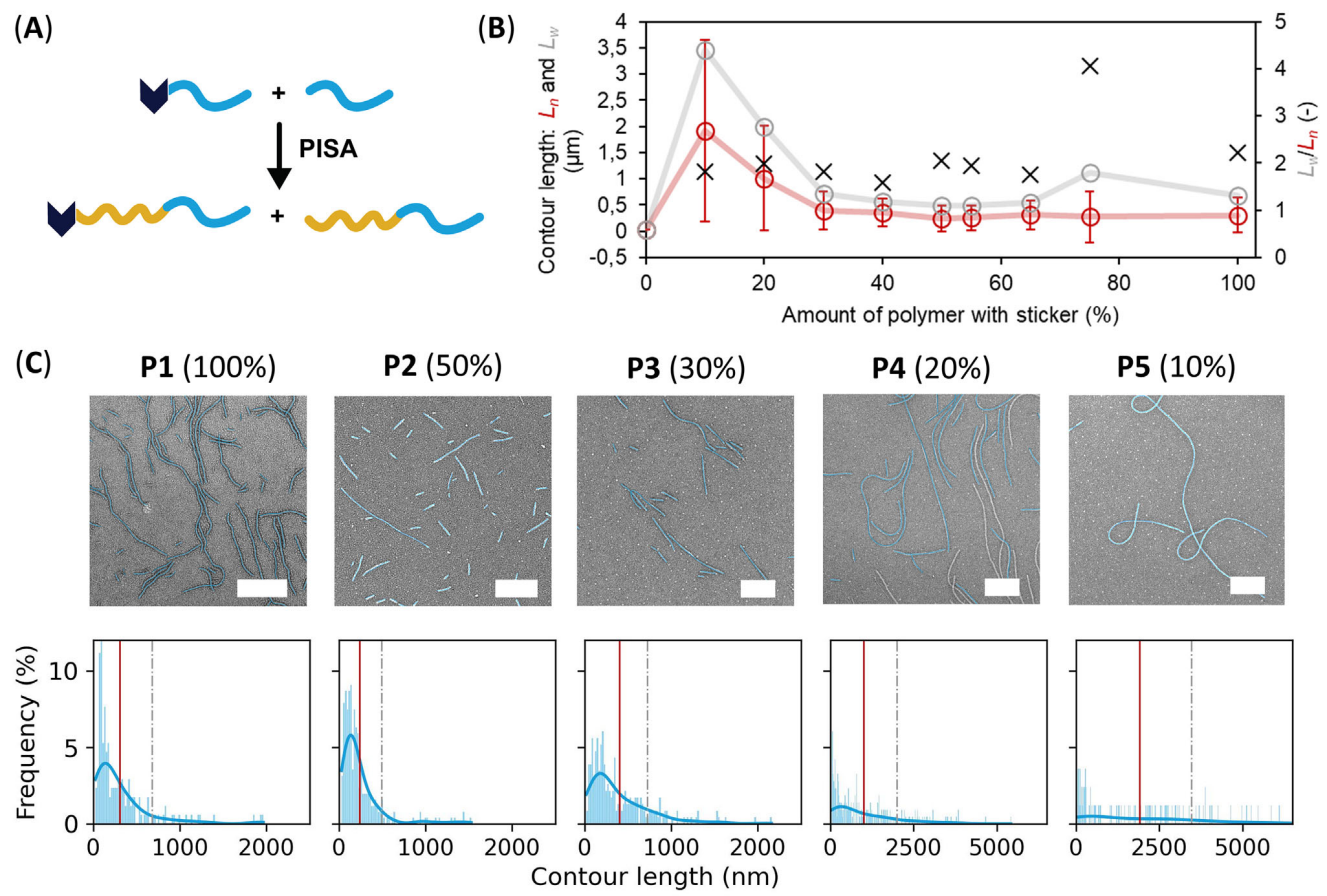
(A) Schematic representation of macroCTA combinations as input for (templated) PISA. (B) Results of image analysis of TEM micrographs of macroCTA combinations ranging from 100% (**P1**) to 0% (**P6**) sticker‐containing polymer, reported as number‐ and weight‐based averages of the contour length (*L*_n_, red line and *L*_w_, gray line). The error bars represent the standard deviation (*σ*) as defined by Equation S3 in the Experimental Section. Black crosses indicate *L*_w_/*L*_n_. For 0% sticker (**P6**), the *z*‐average diameter (*d*_h_ = 30 nm) obtained from DLS is reported instead of *L*_n_ or *L*_w_. (C) Corresponding TEM micrographs and contour length distributions of **P1**‐**P5**. *L*_n_ is marked by the red line, *L*_w_ by the gray dashed lines. Scale bar = 500 nm in all panels.

To test this strategy, we assessed combinations of macroCTAs with and without bis‐urea sticker in different ratios: Briefly, the TTC‐U_2_‐p(DMAc):TTC‐0‐p(DMAc) ratio was varied between 0% and 100 mol% as stated in Table 1. As shown before, the MEA polymerization kinetics from both macroCTAs is nearly identical, ensuring that observed differences in assembly behavior are solely caused by variations in the end‐group ratios. The results of this set of experiments are summarized in Figure 3. In contradiction with our initial hypothesis, we did not observe a continuous decrease in average contour length upon lowering the percentage of sticker‐containing macroCTA. Reducing the sticker content from 100 to 40 mol% had virtually no impact on the length (and dispersity) of the FMs. Surprisingly, significantly longer FMs were found upon further decreasing the fraction of sticker‐containing CTA to 10 mol%, a trend that was reproducibly observed in three independently synthesized batches Figure S11). Under these conditions, an average contour length of approximately *L_n_* = 1.9 ± 1.7 µm was found, while the sample contained FMs of contour lengths exceeding 10 µm (Figure S11). The average width remained basically unchanged for all observed FMs. At 5 and 1 mol%, FMs were scarce, and spherical micelles appeared to be the preferred morphology (not shown). In addition, the persistence length (*P*), a measure of the stiffness of a semiflexible filament defined by the length over which its orientation persists, was determined for the FMs (see Section 4.3 for experimental details). Within experimental error, *L*_n_/*P* remained fairly constant indicating that the flexibility of the micelles does not significantly depend on the initial macroCTA mixing ratio.

### 2.3 The *t*‐PISA Starting Point: Self‐Assembly Behavior of (Mixed) macroCTAs

**FIGURE 4:**
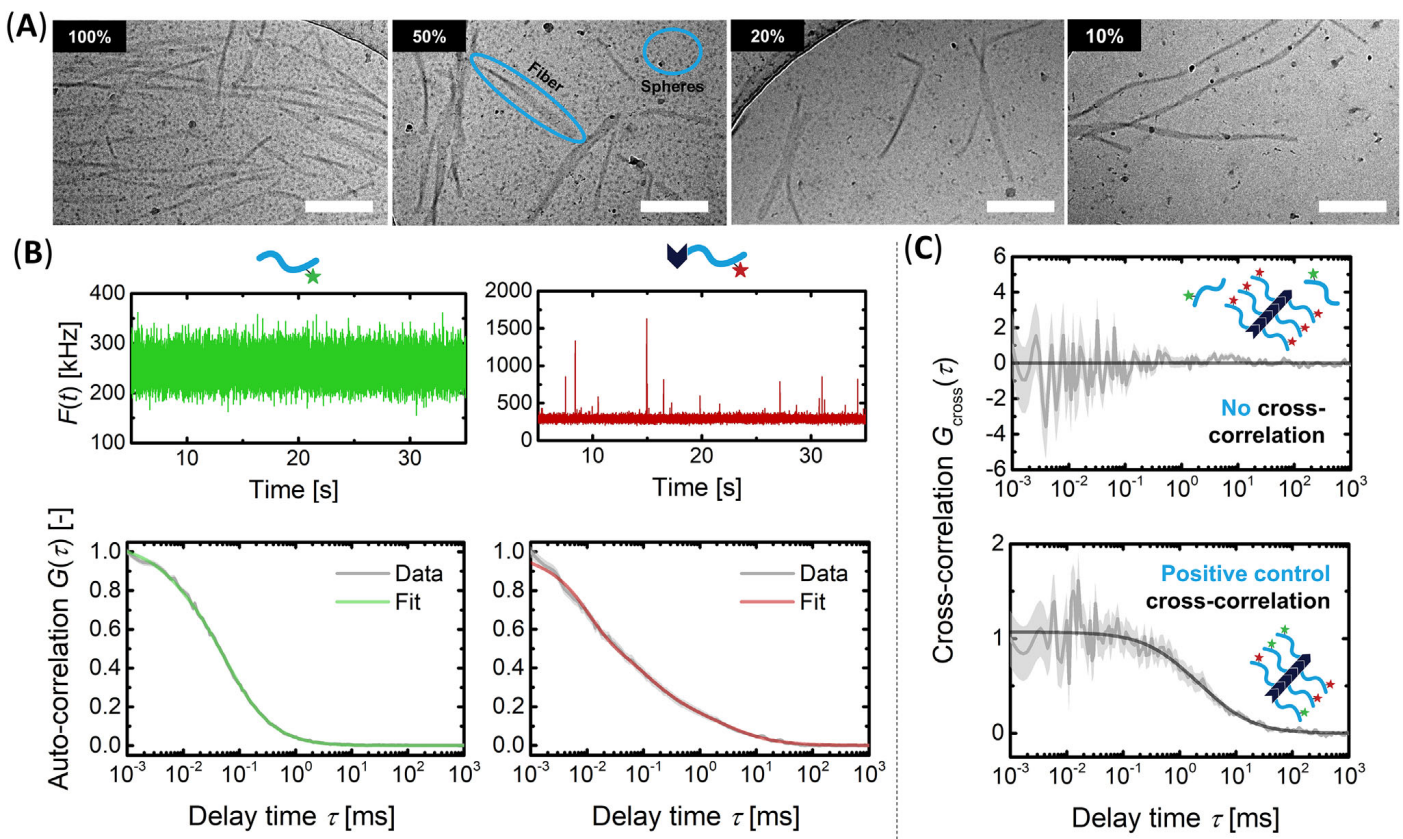
(A) Cryo‐TEM images of macroCTA combinations (100, 50%, 20%, and 10% with sticker, from left to right) dispersed in water (concentration = 9 nmol/mL). Scale bar = 200 nm. (B) Fluorescence fluctuation time traces (*F*(*t*), **top**) and corresponding autocorrelation functions (**bottom**) for a 50:50 mixture of Atto425‐TTC‐0‐p(DMAc) (green) and sulfo‐Cy5‐TTC‐U_2_‐p(DMAc) (red) recorded using excitation wavelengths specific for Atto425 and sulfo‐Cy5 labels. (C) **Top**: Cross‐correlation calculated between the *F*(*t*) traces shown in panel B. No cross‐correlation was found as reflected by the dark gray fitting line. **Bottom**: Positive control of cross correlation for a 50:50 mixture of Atto425‐TTC‐U_2_‐p(DMAc) and sulfo‐Cy5‐TTC‐U_2_‐p(DMAc), where both labels are now incorporated into the sticker‐containing fibers (See cartoon in the bottom right corner).

To elucidate the mechanistic origin of the counterintuitive effect of mixing sticker‐ and non‐sticker‐containing macroCTAs on the FM length, we first investigated the (co‐)assembly behavior of the two macroCTAs. Mellot et al. reported previously that the TTC‐U_2_‐p(DMAc) macroinitiator (i.e. without the hydrophobic MEA block) has the tendency to form fibers, that coexist with spheres upon direct dispersion in water [37]. Cryo‐TEM analysis (Figure 4A, left) confirmed the assembly of TTC‐U_2_‐p(DMAc) into fiber‐ or ribbon‐like structures, coexisting with a relatively large number of small spherical micelles. These micelles are presumably formed when the steric effects of the p(DMAc) outcompetes the directional stacking of the bis‐urea sticker moieties. Surprisingly, the apparent concentration of the spherical micelles seems to decrease with increasing amounts of TTC‐0‐p(DMAc). Furthermore, for mixtures of TTC‐0‐p(DMAc):TTC‐U2‐p(DMAc), the fiber length increases significantly at a ratio of 90:10 (Figure 4A, right), accompanied by a substantial reduction in the apparent number of fibers. Notably, this preassembly behavior parallels the formation of exceptionally long FMs (>10 µm; Figure S11) during PISA at the same macroCTA ratio (Figure 3).

To gain deeper insight into this unexpected result, we used fluorescence (cross) correlation spectroscopy (F(C)CS) to probe the composition of the macroCTA fibers using mixtures of differently labeled macroCTAs. F(C)CS measures fluctuations in fluorescence intensity as a function of time (*F*(*t*)), in a fixed observation volume (*V*_obs_) defined by a focused laser beam that is able to excite the fluorescent molecules present in the sample [38]. The fluctuations arise from diffusion in and out of *V*_obs_. Diffusion coefficients (*D*) can be extracted from the *F*(*t*) traces by calculating an autocorrelation function and fitting this to an analytic model that describes the diffusive processes. In essence, this is analogous to the data processing performed for DLS. In addition to autocorrelation analysis performed in conventional FCS, *F*(*t*) traces obtained for different fluorescent labels simultaneously present in the sample can be cross‐correlated. Essentially, this multicolor equivalent probes how similar the diffusive characteristics of both labels are. A high degree of cross‐correlation indicates that both labels co‐diffuse through the solution because they are present on the same assembly, whereas the absence of cross‐correlation reflects independent Brownian motion.

To enable F(C)CS measurements, both macroCTAs were fluorescently labeled with Atto425 for TTC‐0‐p(DMAc) and with sulfo‐Cy5 for TTC‐U_2_‐p(DMAc). Covalent attachment of these fluorescent tags was achieved through copolymerization of a small fraction of acrylic acid *N*‐hydroxysuccinimide ester (AA‐NHS) with DMAc, followed by amide coupling with primary amine functionalities located on the dyes (see Supporting Information S1.3 and Figure S4). Spectral crosstalk is minimized due to negligible overlap of the excitation (*λ*_ex, Atto425_ = 440 nm; *λ*_ex, sulfo‐Cy5_ = 649 nm) and emission spectra *λ*_em, Atto425_ = 485 nm; *λ*_em, sulfo‐Cy5_ = 662 nm of both labels (see Figure S17 for excitation and emission spectra). This selection of dyes enabled us to independently track and cross‐correlate Atto425‐TTC‐0‐p(DMAc) and sulfo‐Cy5‐TTC‐U_2_‐p(DMAc).

Figure 4B shows the *F*(*t*) traces for Atto425‐TTC‐0‐p(DMAc) in green, and sulfo‐Cy5‐TTC‐U_2_‐p(DMAc) in red recorded in a 50:50 mixture of both macroCTAs (Figure 4A, second image), which revealed a distinctly different appearance. Rapid and rather uniform fluctuations are observed for the Atto‐labeled species, whereas the sulfo‐Cy5‐TTC‐U_2_‐p(DMAc) trace reveals a spiky, irregular pattern. Qualitatively, this can be directly related to the solution state of both species. The rapid and uniform fluctuation of Atto425‐TTC‐0‐p(DMAc) translates to homogeneously labeled and fast diffusing species expected for relatively small molecules free in solution. This assessment is in agreement with the autocorrelation analysis (Figure 4B, bottom left), which yielded *D* = 143 ± 3 µm^2^/s. This value closely matches that of Atto425‐TTC‐0‐p(DMAc) free in solution (see Table S4, *D* = 150 ± 20 µm^2^/s) and correspond to a hydrodynamic radius *R*_h_ ≈ 1.5 nm, which is a reasonable value for a soluble polymer with a relatively low DP (≈ 31).

On the contrary, the unevenly intense fluorescent signals measured for sulfo‐Cy5‐TTC‐U_2_‐p(DMAc) relate to larger assemblies with slow diffusive dynamics and a higher fluorescent brightness. The corresponding autocorrelation function could only be fitted with a two‐component model yielding a fast (119 ± 23 µm^2^/s) and slower (2.4 ± 0.4 µm^2^/s) diffusive species. These values for *D* have moderate quantitative value due to the limited sampling of the slow diffusion species (i.e., small number of fluorescent spikes) over the course of a measurement and strong impact that the fluorescent spikes have on the resulting autocorrelation function. Nevertheless, the obtained values are again analogous to those obtained for a solution containing only sulfo‐Cy5‐TTC‐U_2_‐p(DMAc) instead of a 50:50 mixture (see Table S4).

From these results it appears that the sticker‐ and non‐sticker‐containing macroCTAs do not co‐assemble into mixed aggregates. The observed spherical and fiber‐like assemblies in cryo‐TEM (Figure 4A) comprise exclusively the sticker‐containing macroCTA while TTC‐0‐p(DMAc) remains molecularly dissolved (not visible with cryo‐TEM). This conclusion is further supported by the complete absence of any cross‐correlation between the Atto425 and sulfo‐Cy5 labeled channels (Figure 4C, top). To verify that simultaneous incorporation of Atto425‐ and sulfo‐Cy5‐labeled species in the fiber‐like assemblies would result in a measurable degree of cross‐correlation, a control FCCS experiment was performed on a sample in which a part of TTC‐U_2_‐p(DMAc) was labeled with Atto425 and a part with sulfo‐Cy5. As expected, this positive control led to significant cross‐correlation (Figure 4C, bottom) and a diffusion coefficient in close agreement with the value obtained for only sulfo‐Cy5‐TTC‐U_2_‐p(DMAc).

**FIGURE 5:**
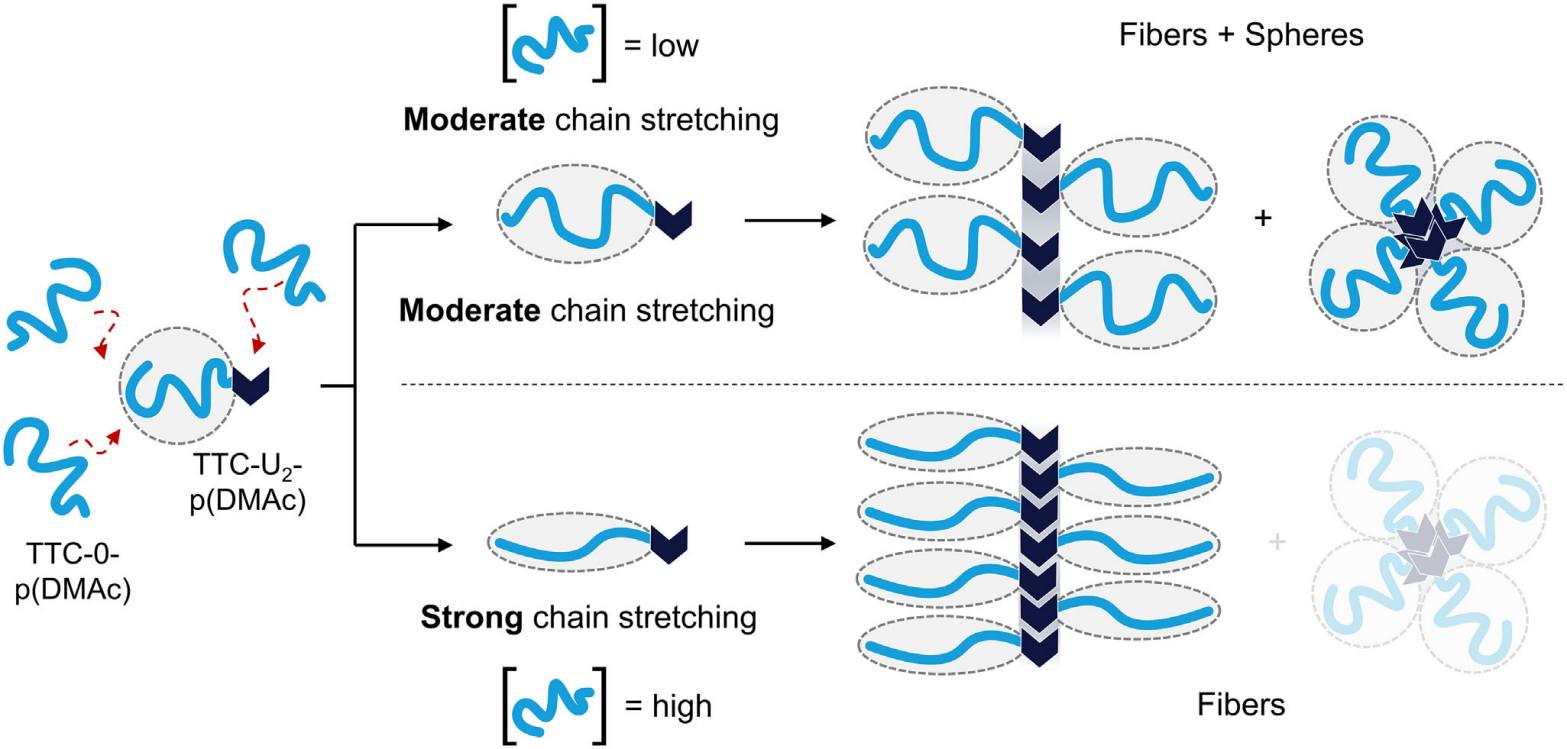
Schematic representation of the hypothesized assembly behavior of sticker‐containing macroCTAs (TTC‐U_2_‐p(DMAc)) in the presence of macroCTAs without the sticker end‐group (TTC‐0‐p(DMAc)). **Bottom**: High concentrations of TTC‐0‐p(DMAc) lead effectively to an enhanced p(DMAc) chain stretching, exposure of the sticker and thus enhanced fiber formation. **Top**: In the absence or at lower TTC‐0‐p(DMAc) concentrations, chain stretching is less pronounced. In this situation, TTC‐U_2_‐p(DMAc) adopts (random) coil conformations, causing steric congestion that promotes the formation of spherical micelles.

Having established that both macroCTAs do not co‐assemble does not explain the apparent increase in fiber length upon lowering the fraction of sticker‐containing CTA at the expense of the spherical micelles (Figure 4A). Intuitively, one could expect the opposite trend where the assemblies become shorter. However, the conformational freedom of the p(DMAc) chains should be taken into consideration. For thermodynamic reasons, the polymer chains preferentially adopt a (random) coil conformation rather than an extended chain with a well‐exposed chain end. The steric congestion impedes 1D stacking of the bis‐urea sticker and promotes the formation of spherical micelles having the rather hydrophobic sticker moiety more closely but randomly stacked in their core (Figure 5, top).

The situation changes upon addition of sticker‐free TTC‐0‐p(DMAc), which effectively is a soluble homopolymer with the same chemical structure as the corona‐forming blocks in the micellar assemblies. Several studies have demonstrated that dissolved corona‐forming homopolymers can affect micellar stability and exchange dynamics through weak penetration into the micellar corona. This interaction leads to changes in aggregation numbers and micellar core sizes, depending on the affinity between the homopolymers and the corona chains [39, 40]. Such changes in micelle size and aggregation number, as observed by simulations and neutron scattering experiments, were already apparent at relatively low homopolymer concentrations; comparable to the TTC‐0‐p(DMAc) concentrations present in mixtures containing only 10%–20% sticker‐containing macroCTAs. Extending these concepts to systems comprising sticker‐containing macroCTAs, we hypothesize that higher concentrations of TTC‐0‐p(DMAc) promote more extended corona conformations in order to accommodate the weak, transient insertion of dissolved homopolymers. The formation of extended coronas would, in turn, favor the development of fiber‐like structures, as the bis‐urea stickers can more readily stack through directional hydrogen‐bonding (Figure 5, bottom). Additionally, the dissolved TTC‐0‐p(DMAc) could acts as macromolecular depletants that promotes supramolecular stacking of the bis‐urea moieties. As reported by Van der Tol et al. this mechanism requires the depletant concentrations close to the overlap concentration (c*) [41]. Estimating c* (Supporting Information 5, ≈ 48 mg/mL) indicates that the total, experimentally used macroCTA concentration lies approximately a factor of two below c*. This suggest a potentially weak contribution of depletion forces to macroCTA assembly in samples containing substantially more TTC‐0‐p(DMAc) than TTC‐U2‐p(DMAc). In agreement with the F(C)CS data, both stretching and depletion mechanisms do not require co‐assembly of both macroCTAs (Figure 4C, top).

### 2.4 During a *t*‐PISA Reaction: Mechanism of FM Formation

**FIGURE 6:**
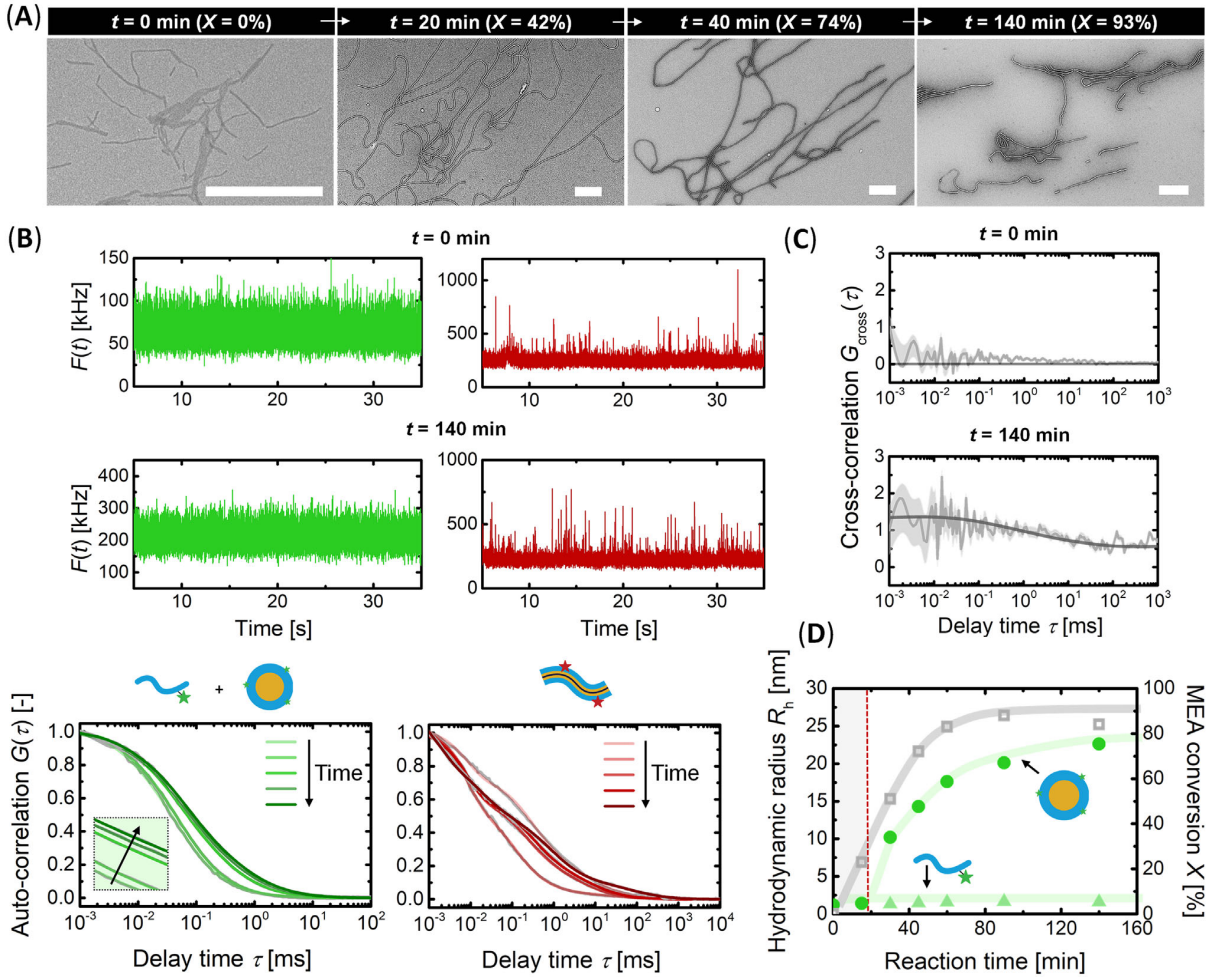
(A) Dry‐state TEM images of evolution (from left to right) of fiber formation during *t*‐PISA for a reaction equivalent to **P5**. Scale bar = 1 µm. Please note: the apparent visual differences of the particles are related to variation in effectiveness of the staining procedure. (B) **Top**: Fluorescence fluctuation time traces (*F*(*t*)) for diluted reaction mixtures of **P5** containing Atto425‐TTC‐0‐p(DMAc) (green, left) and sulfo‐Cy5‐TTC‐U_2_‐p(DMAc) (red, right) measured on samples taken after a reaction time 0 and 140 min. **Bottom**: Autocorrelation as a function of the reaction time for the Atto425 excitation channel (left) and sulfo‐Cy5‐channel (right). The green‐highlighted zoom‐in of the Atto425 curves shows a gradual shift of the characteristic delay time for the correlation function to longer delay times as indicated by the arrow. This trend was not observed for the correlation functions in the sulfo‐Cy5 channel. (C) Cross‐correlation calculated between the *F*(*t*) traces shown in Panel (B). Only the sample after 140 min showed minor degrees of cross‐correlation. (D) MEA conversion for **P5** a (gray squares) and evolution of the hydrodynamic radius *R_h_* of Atto425‐labelled species as a function of reaction time. *R_h_* values were calculated based on diffusion coefficients (*D*) extracted from the autocorrelation fits shown in Panel (B). Either a 1‐component (left of the red dotted line) or a 2‐component fit (right of the red dotted line) was used. The green circles correspond to (forming) spherical micelles, whereas the triangles represent freely dissolved Atto425‐labeled (block‐co)polymers. Lines are drawn to guide the eye.

Next, we investigated how the preassembled configuration of macroCTAs (described in the previous section) evolves during the subsequent PISA reaction. A sample containing a 10:90 ratio of TTC‐U_2_‐p(DMAc):TTC‐0‐p(DMAc) was selected (**P5**), as this composition produced the longest preassembled and post‐polymerization fibers. Taking aliquots and evaluating the FM evolution as a function of reaction time with dry‐state TEM revealed that the initial macroCTA fibers persist during polymerization (Figure 6A). The fibers become more distinct during the reaction and their width increases with monomer conversion. Quantitative assessment of the fiber lengths throughout the PISA process was not attempted due to limited statistics and difficulties associated with tracing extremely long FMs. Nevertheless, fiber length does not increase continuously with monomer conversion, as fibers are observed at all stages of the *t*‐PISA process [31, 32].

F(C)CS was again used to monitor the fate of the BCPs derived from both sticker‐ and non‐sticker containing macroCTAs. For this set of experiments, TTC‐U_2_‐p(DMAc) labeled with sulfo‐Cy5 and TTC‐0‐p(DMAc) labeled with Atto425 were used. *F*(*t*) traces for both fluorescent channels at *t* = 0 and 140 min, corresponding to 0 and approximately 90% MEA conversion, respectively, are shown in Figure 6B. These traces are representative for all measured time points. Although the fluorescence fluctuation signatures (Figure 6B) appear visually unchanged over time, the degree of cross‐correlation between the Atto425‐ and sulfo‐Cy5‐labeled species shows a small but significant increase over the course of the PISA process (Figure 6C). The low signal‐to‐noise level for the cross‐correlation measured after the PISA reaction reveals a low degree of co‐assembly between the sticker and non‐sticker containing BCPs compatible with potential insertion of some non‐sticker containing polymers into the polymeric fibers. Considering that the fluorescence fluctuations of the Atto425‐labeled non‐sticker containing BCPs remain homogeneous and fast, insertion is however not the primary fate of this species. This observation is also consistent with the rather constant FM flexibility regardless of the TTC‐U_2_‐p(DMAc):TTC‐0‐p(DMAc) ratio as the FMs consist in all cases of nearly only sticker‐containing polymers (Figure S15).

To gain more insight in the distribution of this species in the sample, autocorrelation analysis was performed on the Atto *F*(*t*) traces for each time point. The autocorrelation functions show a gradual increase to longer delay times as reaction time progresses (Figure 6B, bottom left). Fitting the correlation curves to extract values for *D* and converting these to hydrodynamic radii (*R*_h_) using the Stokes–Einstein equation, revealed that the Atto425‐labels were distributed over freely dissolved polymers (Figure 6D, triangles) that coexisted with small assemblies (Figure 6D, circles). These assemblies, most probably spherical micelles, increase in size as MEA is polymerized and reach a final *R*_h_ of 23 nm, which is in good agreement with the spherical micelles formed in PISA mixtures containing solely TTC‐0‐p(DMAc) macroCTAs (Table 1, **P6**). Although not always clearly visible on (dry‐state) TEM images, this result does confirm that the FMs coexist with spherical micelles which are the primary depot for the non‐sticker containing BCPs. The *R*_h_ of the dissolved polymers remained rather constant, which could be related to a fraction of TTC‐0‐p(DMAc) macroCTA that is not effectively chain extended or the coexisting concentration of free BCPs equal to the critical micelle concentration. The autocorrelation curves in the sulfo‐Cy5 channel show the presence of large assemblies, consistent with the presence of long wormlike structures throughout the reaction. As discussed before, the strongly irregular *F*(*t*) traces make quantitative assessment of these curves not trustworthy. In essence, the preassembled state established by mixing sticker‐ and non‐sticker‐containing macroCTAs (Figure 5, top) acts as a structural blueprint for the morphologies produced during *t*‐PISA, giving rise to long fibers alongside spherical micelles.

**FIGURE 7:**
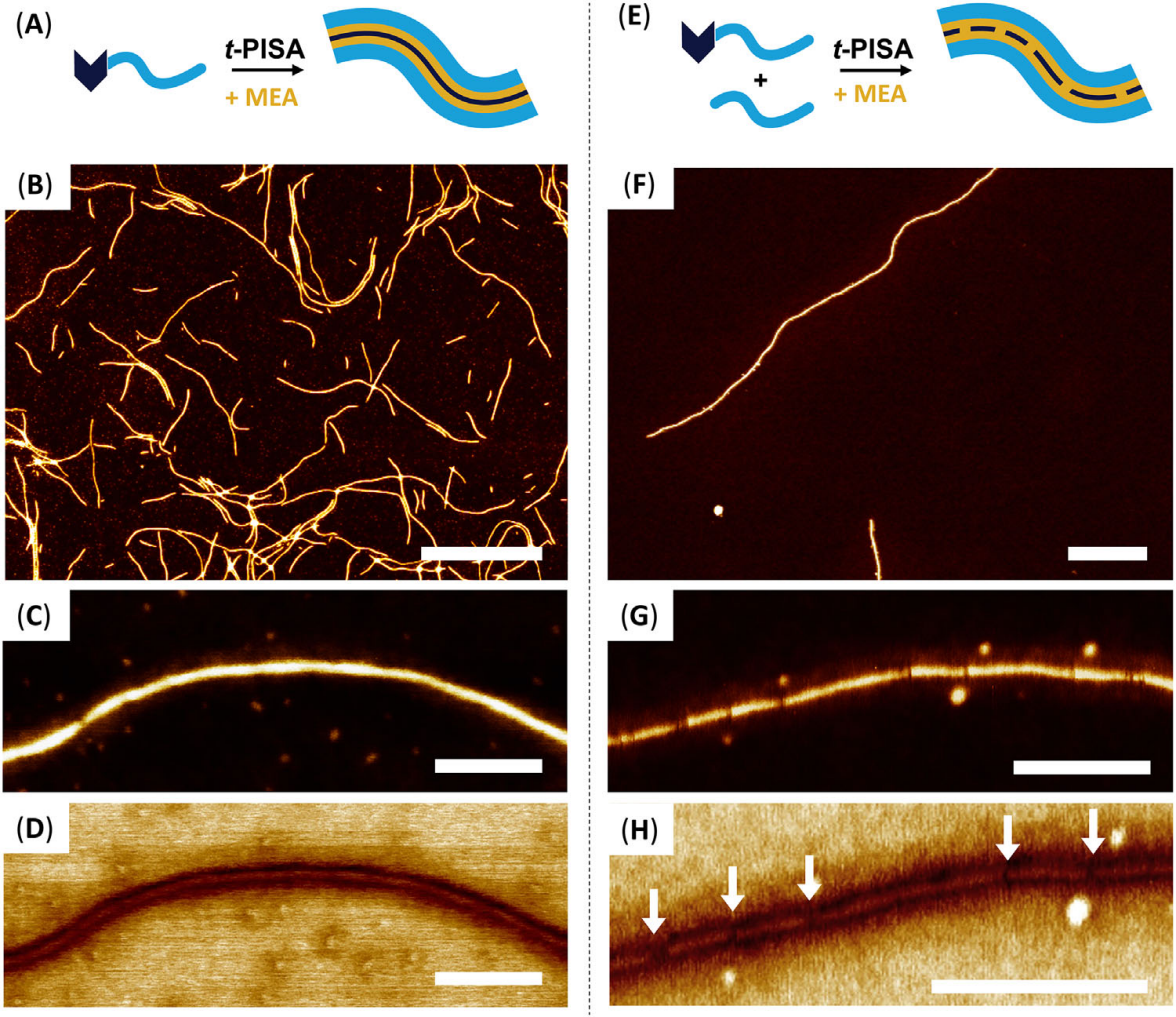
Schematic representation of FMs obtained from *t*‐PISA reactions using (A) solely sticker‐containing macroCTAs (TTC‐U_2_‐p(DMAc) (**P1**) or (E) a mixture of non‐sticker: sticker macroCTAs (TTC‐0‐p(DMAc):TTC‐U_2_‐p(DMAc) with a 90:10 ratio (**P5**). AFM height images of the FMs at (B,F) lower magnification (scale bar = 500 nm) and (C,G) zoomed in (scale bar = 100 nm) for **P1** and **P5**, respectively. The vertical scale varies from 0 to 8 nm for each height image. Derjaguin–Muller–Toporov (DMT) elastic moduli derived from FM shown in (D) panel C and (H) panel G. Lighter colors correspond to stiffer features. The arrows in panel H highlight the soft discontinuities along the contour length.

To substantiate the mechanistic insights provided by F(C)CS, AFM imaging was performed, motivated by earlier work by Mellot et al. [31, 32] on comparable FMs. This study demonstrates that AFM can differentiate between the stiffer bis‐urea‐containing core and the softer corona‐forming block. This capability allowed us to hypothesize that the insertion of non‐sticker BCP domains into FMs could be visualized as localized softening, arising from disruption of the bis‐urea stacks. Probing topological features with AFM for **P1** (100% TTC‐U_2_‐p(DMAc)‐*b*‐p(MEA) and **P5** (TTC‐0‐p(DMAc)‐*b*‐p(MEA)): TTC‐U_2_‐p(DMAc)‐*b*‐p(MEA) = 90:10) (Figure 7, see Section S2.8 for experimental details) gave images that show striking resemblance with the dry‐state TEM analysis performed earlier (Figures 7B and F). Please note that the overall concentration of extremely long FMs in **P5** is significantly lower compared to that in **P1**, making it challenging to capture multiple FMs in a single image. To ensure the shown images are a fair representation of the sample, additional AFM images are included in Supporting Information Section S6. The FM widths were extracted from indentation topographic maps.

For each sample, at least 40 height profiles were analyzed. The widths were in the range of 35–45 nm (39 ± 3 nm for **P1** and 40 ± 5 nm for **P5**), which are in reasonable agreement with the values obtained form dry‐state TEM (Figure 2). Imaging **P1** and **P5** at a higher magnification revealed striking differences between the FMs. Whereas, **P1** showed nearly exclusively homogeneous fiber‐like structures (Figure 7C), the **P5** FMs appear all segmented (Figure 7G and Supporting Information Section S6 for additional images). This segmentation is further emphasized in the elastic moduli derived from the indentation maps. **P1** shows the expected uniform mechanical properties along the contour length of the fiber (Figure 7D). The relatively stiff core (6.5 ± 1 nm) is flanked by two darker regions, which correspond to the p(DMAc) stabilizing corona. For **P5**, these relatively stiff core segments are longitudinally separated by darker, and therefore mechanically softer, sections. Provided that the bis‐urea stickers are the major contributor to the core‐stiffness, these soft connecting segments are likely the result of insertion of BCPs that do not carry the sticker. We hypothesize that segmented incorporation is favored over homogeneous block copolymer insertion because integration into the supramolecular stack requires local disruption of bis‐urea hydrogen bonding. Once a non‐sticker‐containing polymer has overcome this energetic barrier and created a defect within the stack, subsequent polymers can insert with reduced energetic cost, preferentially occupying adjacent positions. Based on AFM, no quantitative assessment can be made on the relative number of non‐sticker‐containing polymers and whether the weaker areas are completely devoid from bis‐urea stickers, although FCCS indicates only moderate incorporation.

### 2.5 After a *t*‐PISA Reaction: Obtaining Pure FMs

As both F(C)CS and AFM indicated the coexistence of the target FMs with relatively high concentrations of spherical micelles, we sought to isolate the FMs via centrifugation. A stepwise centrifugation protocol with gradually increasing rotational speeds was employed to achieve optimal separation of fibrous and spherical micelles while avoiding shear‐ or flow‐induced scission of the elongated fibers. A detailed description of the centrifugation protocol and data analysis is provided in Supporting Information Section S1.6 and S7. DLS measurements confirmed effective separation, while TEM analysis showed that the isolated FMs withstand the applied centrifugal forces (see Figure S22 and S23). The ability to isolate FMs enhances the applicability of the previously described strategy and enables systematic evaluation of the performance of exceptionally long FMs across diverse application areas without interference from morphological impurities.

### 2.6 Controlling the Length in the Opposite Direction: Sonication‐Induced Worm Scission

**FIGURE 8:**
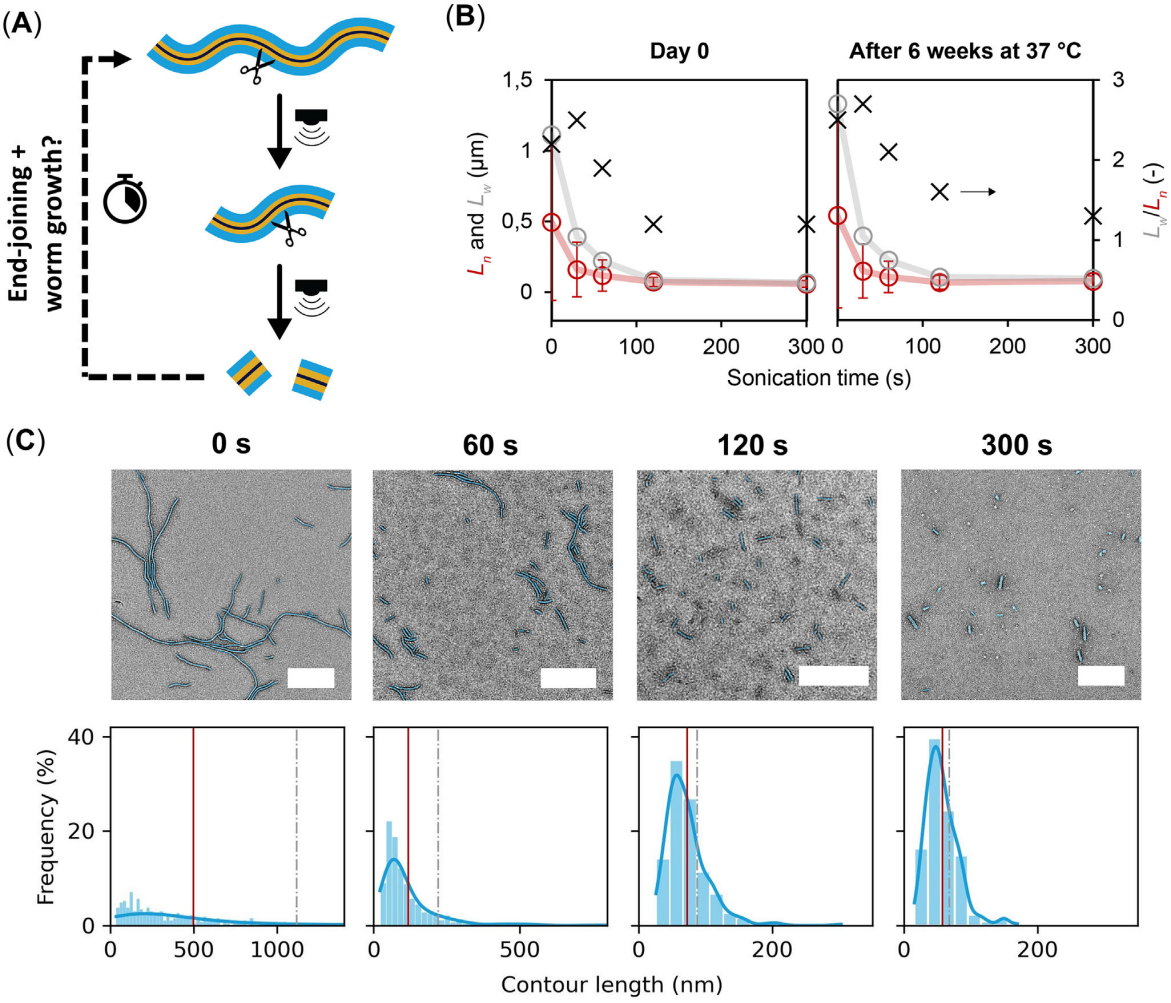
(A) Schematic representation of sonication‐induced FM scission. (B) Number‐, weight‐based averages of the contour length (*L*_n_, red line and *L*_w_, gray line) and length polydispersities (crosses, *L*_n_/*L*_w_) of FMs as a function of sonication time. Values were extracted from TEM image analysis directly after sonication (day 0, left) and after storage of those sonicated samples for 6 weeks at 37 °C with gentle agitation on a roller (right). The error bars represent the standard deviation (*σ*) as defined by Equation S3 in the Materials & Methods Section. (C) Individual TEM micrographs and corresponding contour length distributions (day 0) as function of the sonication time. *L*_n_ is marked by the red line, *L*_w_ by the gray dashed line. Scale bar = 500 nm. Please note that TEM analysis was performed on highly diluted samples to reliably trace individual FMs. A multitude of TEM images were used to trace statistically reliable contour length distributions.

Having established a mechanistic understanding of the macroCTA mixing strategy and showing that this approach could be leveraged for the synthesis of extremely long FMs, a different strategy, namely post‐synthesis sonication, was explored to shorten the FMs and broaden the spectrum of achievable aspect ratios (Figure 8A). As previously reported, sonication imposes mechanical stresses on the FMs [42]. Longer micelles are more susceptible to these forces and therefore likely to be cleaved first, leading to not only a shortening of the average contour length but also a narrowing of the length distribution.

To assess the translatability of this approach to the templated FMs, dilutions of TTC‐U_2_‐p(DMAc)_30_‐*b*‐p(MEA)_90_ (**P1**) were subjected to probe tip sonication for different times (30 up to 300 s, non‐treated control = 0 s sample). Samples were cooled in an ice bath during sonication to prevent excessive dissipative heating (see Figure S26 for measured temperature profile during sonication). **P1** was selected for this study to circumvent interference of spherical micellar side‐products without the need for FM isolation by centrifugation. Although not quantitative in terms of size, a clear shift in the correlation curves obtained from DLS indicated sonication‐time dependent shortening, with the most significant change occurring in the first 30 s (Figure S24). Quantification of the shortening effect was conducted with dry‐state TEM and image analysis as described previously. As indicated in Figure 8B, the average contour length (*L*_n_) reduced from 495 to 160 nm within the first 30 s of sonication, corresponding to a 68% reduction. After 300 s, the total reduction appears to plateau at *L*_n_ ≈ 58 nm (about 88% reduction). Accompanied with the shortening also the anticipated sharpening of the contour length distribution was observed (Figure 8C).

Sonication potentially creates cut‐off ends that expose the hydrophobic core of the micelles, providing a driving force for end‐joining and thus regrowth. To determine the long‐term stability of the particles, the sonicated samples were split up into four different storage conditions and TEM images were taken again after 3 and 6 weeks: (***i***) at 4°C, no agitation, (***ii***) 21°C, no agitation, (***iii***) 21°C, agitated on a roller, and 37°C, agitated on a roller (Supporting Information Section S8). Conditions with increased temperature and agitation may facilitate potentially occurring end‐joining/worm growth. Nevertheless, image analysis indicated good particle stability, as only negligible changes in the morphology, contour length distribution and polydispersity were found for the shortened particles, regardless of the storage condition (see Figure 8B for assessment of storage condition (***iv***) after 6 weeks as a representative indication of storage stability). Therefore, sonication proved to be a fast and robust tool to shorten FMs from highly anisotropic to nearly spherical morphologies, which remain stable over time.

## 3 Conclusion

In this study, we have explored two strategies to tailor the contour length of FMs synthesized via a *t*‐PISA procedure. The templating originates from macroCTAs carrying supramolecular bis‐urea stickers as end‐group. These stickers direct the self‐assembly process of the (block co‐)polymers into a 1D fashion, generating FMs. Mixing in macroCTAs that do not contain this sticker was anticipated to result in shorter micelles by end‐capping. Unexpectedly, this strategy resulted in the opposite effect, where FM lengths increase upon lowering the fraction of sticker‐containing macroCTAs yielding highly anisotropic micelles with lengths up to 10 µm. Detailed cryo‐TEM and F(C)CS experiments revealed that both classes of macroCTAs (with and without sticker units) and their derived BCPs do not readily co‐assemble, but suggest that the non‐sticker macroCTA promotes the fiber formation of its sticker‐containing counterpart. The longer initial fibers formed by the sticker‐containing macroCTA carry over to the PISA process to yield extremely anisotropic FMs. Relying on post‐PISA sonication as a second strategy to control the contour length was effective in shortening the FMs down to near spherical morphologies.

Taken together, both the macroCTA mixing and sonication strategies allow tuning of the contour lengths of these micelles over a broad range of anisotropies. Considering that the resulting FMs are identical in thickness and chemistry, we believe that these strategies contribute to the realization of particle libraries with controllable dimensions and aspect ratios. These libraries could in turn be used to establish more detailed structure‐performance relationships and facilitate rational design of wormlike micelles in relevant application areas, for example, drug delivery.

## Conflicts of Interest

The authors declare no conflicts of interest.

## Supporting information




**Supporting File**: smll74648‐sup‐0001‐SuppMat.pdf.

## Data Availability

The data that supports the findings of this study are available in the supplementary material of this article
