# Supplementary material for: Tuning the Length of Filomicelles Prepared Via Templated Polymerization‐Induced Self‐Assembly
Source: Small. 2026 Jul 17;22(51):e74648. doi: 10.1002/smll.74648 (PMC13568845; doi:10.1002/smll.74648)
Supplement: Supplementary file 1 [file SMLL-22-e74648-s001.pdf]

Supporting Information

Tuning the length of filomicelles prepared via templated polymerization-induced self-assembly

*Eleonora G. Hochreiner[1], Wiktoria Pioruńska[1], Gaëlle Mellot[2], Bence Fehér[3,‡], Jean-Michel Guigner[4], Viktoriia Drebezghova[5], Christophe Sinturel[5], Cornelus F. van Nostrum[1], Tina Vermonden[1], Jutta Rieger[2], Bas G. P. van Ravensteijn[1]**

[1] Division of Pharmaceutics, Utrecht Institute for Pharmaceutical Sciences, Faculty of Science, Utrecht University, Utrecht 3584 CG, The Netherlands.

[2] Sorbonne Université, CNRS, Institut Parisien de Chimie Moléculaire (IPCM) F-75005 Paris, France.

[3] Department of Chemical Engineering and Chemistry, Institute for Complex Molecular Systems, Eindhoven University of Technology, 5600 MB, Eindhoven, The Netherlands.

[4] Sorbonne Université, CNRS, Institut de Minéralogie, de Physique des Matériaux et de Cosmochimie (IMPMC), 75252, Paris, France

[5] Université d'Orléans, CNRS, ICMN, UMR 7374, Orléans, France.

*To whom correspondence should be addressed: b.g.p.vanravensteijn@uu.nl

‡ Current affiliation: Institute of Biophysics and Radiation Biology, Semmelweis University, Budapest, Hungary.

CONTENT

S1) **Materials & methods**……………………………………………………………………..3

S2) **Characterization techniques**………………………...………………………………..8

S3) **Polymer synthesis and characterization**……………………………………………….15

S4) **TEM image analysis**………………………………………………………………….21

S5) **Overlap concentration (*c**) estimation of TTC-0-p(DMAc)**…………………………..27

S6) **Characterization of macroCTA preassemblies with DLS and F(C)CS**……………...28

S7) **Additional AFM images**…………………………………………………………...31

S8) **Isolation of FMs via centrifugation**……………………………………………………33

S9) **Shortening via sonication and storage stability**……………………………….…34

S1) Materials & methods

S1.1. Materials. Trithiocarbonate (TTC)-based CTAs TTC-0 and TTC-U_2 (**Figure 1B** in **Main Text**) were synthesized based on the procedure reported by Mellot et al.[1] *N*,*N*-dimethylformamide (DMF, peptide synthesis grade) was obtained from Biosolve. Inhibitor removers (beads consisting of basic alumina, for removing hydroquinone and monomethyl ether hydroquinone (MEHQ)), 1,3,5-trioxane (≥ 99%), 2,2′-azobis(2-methylpropionamidine) dihydrochloride (V-50, 97%), 2,2′-azobis(2-methylpropionitrile) (AIBN, 98% or purum, ≥ 98.0% (GC)), acrylic acid *N*-hydroxysuccinimide ester (AA-NHS), deuterated dimethyl sulfoxide (DMSO-d_6, 99.9 atom% D), and deuterated chloroform ($CDCl_3$, 99.8 atom% D) were purchased from Sigma Aldrich/Merck. Deuterated acetone ($(CD_3)_2CO$, 99.8 atom% D) was obtained from Carl ROTH. *N*,*N*-Dimethyl acrylamide (DMAc, 99%, contains MEHQ as inhibitor) and 2-methoxyethyl acrylate (MEA, stabilized with MEHQ; > 98.0 % (GC)) were obtained from TCI Europe N.V.. Sulfo-cyanine5 amine (sulfo-Cy5 amine) was purchased from Lumiprobe GmbH, Atto425 amine from ATTO-TEC GmbH, and SPI-Chem™ uranyl acetate (depleted uranium) from Structure Probe Inc. The water used in all dispersion polymerizations was purified with a Milli-Q purification system (18.2 MΩ·cm at 25 °C) while deionized water was used for dialysis. Monomers (DMAc and MEA) were individually filtered over a column of inhibitor removers before each use. If required, DMF was dried overnight with molecular sieves (4 Å). All other chemicals were used as received unless stated otherwise.

S1.2. Synthesis of TTC-U_2-p(DMAc) and TTC-0-p(DMAc) macroCTAs. TTC-based CTAs (0.18 mmol; for TTC-0: 50 mg; for TTC-U_2: 115 mg; **Figure S1** – **S2** for ^{1}H-NMR spectra) and 1,3,5-trioxane (19 mg, 0.22 mmol) were placed in a Schlenk tube. A stock solution of AIBN (12 mg) in DMF (22 mL) was prepared, and 2.65 mL (containing 8.6 µmol of AIBN) was added to the

Schlenk tube with the CTA. Finally, freshly filtered DMAc (0.78 mL, 7.57 mmol) was added. The mixture was deoxygenated by purging with nitrogen for 30 min while submerged in an ice bath, before starting the polymerization by heating the reaction mixture to 70 °C in an oil bath. The monomer conversion (based on the intensity of the monomer signal at δ = 5.5 ppm) was determined via ^{1}H-NMR in $CDCl_3$ using 1,3,5-trioxane (δ = 5.0 ppm) as an internal standard. After 75 min, the reaction was quenched by cooling to RT and exposure to air. The macroCTA was purified via dialysis (1 kDa MWCO RC membrane tubing) against water (2 L) for 48 h, with water changes after 12 and 24 h. Finally, the product was isolated as a pale yellow solid via freeze drying and characterized by ^{1}H-NMR (in DMSO-d_6) and SEC.

S1.3. Synthesis of fluorescently labelled TTC-U_2-p(DMAc-*co*-AA-NHS) and TTC-0-p(DMAc-*co*-AA-NHS) macroCTAs. To label the macroCTAs with either sulfo-Cy5 amine or Atto425 amine, first AA-NHS (10 mol% of the total monomer content) was copolymerized with DMAc, followed by coupling of the dye amine to the NHS groups via formation of an amide bond (see **Figure S4**). Subsequent dialysis in water purified the macroCTA and hydrolyzed remaining NHS groups prior to isolation via freeze drying.

In a 4 mL glass vial with septum, the CTA (0.054 mmol; either 15 mg TTC-0 or 35 mg TTC-U_2) was combined with AA-NHS (39 mg, 0.23 mmol) and 1,3,5-trioxane (6 mg, 0.07 mmol). A stock solution of AIBN (4.3 mg) in 8.1 mL anhydrous DMF was prepared, and 800 µL of this solution (containing 2.6 µmol of AIBN) was added to the vial. Lastly, DMAc (215 µL, 2.1 mmol) was added to the heterogeneous mixture before deoxygenating the reaction mixture by purging with N_2 for 20 min. The reaction mixture was heated at 70 °C for 105 min. The DMAc conversion was determined with ^{1}H-NMR (in DMSO-d_6). Monitoring the AA-NHS conversion during the polymerization was not feasible due to its high reactivity under the applied reaction conditions and

low intensity of the vinyl AA-NHS signal. However, near-quantitative incorporation is inferred from the negligible residual vinyl signals observed post-polymerization.

For the labelling, the reaction mixture from the previous step (5 µL) was mixed with 2 µL of Et_3N in anhydrous DMF (18 µL Et_3N with 982 µL DMF) and 25 nmol of dye amine in anhydrous DMF (25 µL) and left to react at ambient conditions on a roller bench for 14 h while protected from light. The mixture was transferred to a dialysis cup (MWCO 2 kDa) and extensively dialyzed against deionized water for 48 h prior to freeze-drying. The colored solid was redissolved in Milli-Q water (200 µL) and stored in the fridge until further use. The labeled macroCTAs are referred to as Atto425/sulfo-Cy5-TTC-U_2/0-p(DMAc).

S1.4. RAFT dispersion polymerization of MEA. In a typical experiment, TTC-0-p(DMAc) and/or TTC-U_2-p(DMAc) (in total 7.1 µmol, see **Table 1** in **Main Text** for details) were combined with 1,3,5-trioxane (8 mg, 0.09 mmol) in a 4 mL vial with a septum-containing cap. A stock solution of V-50 (6.9 mg) in 28.8 mL water was prepared, and 815 µL of this solution (containing 0.7 µmol of V-50) was added to the vial. This mixture, as well as freshly filtered MEA in a separate vial were deoxygenated by purging with nitrogen for 20 min while in a precooled aluminum vial block. MEA (90 µL, 0.70 mmol) was added, and the polymerization was started by heating the reaction mixture to 60 °C in a preheated vial block. The monomer conversion was determined via ^{1}H-NMR either in DMSO-d_6 or acetone-d_6 using 1,3,5-trioxane (both in DMSO-d_6 and acetone-d_6: δ = 5.1 ppm) as an internal standard and was reported based on the intensity of the monomer signal at δ = 5.9 ppm. For monitoring the polymerization kinetics, samples were taken from the reaction mixture at regular time intervals and quenched by exposure to air, while maintaining an inert atmosphere in the reaction vial. After 140 min, the polymerization was quenched by immersing the vial in iced water and exposing the mixture to air. To purify the polymers, the

reaction mixture was extensively dialyzed against water for 48 h using 1 kDa MWCO RC membrane tubing. The solid content was determined gravimetrically in triplicates. The particle batch used for the sonication experiments was prepared on double the scale. No significant variations in the final particle size and morphology were observed.

In case of the sample containing a 90:10 ratio of TTC-0-p(DMAc):TTC-U_2-p(DMAc) which was studied by fluorescence (cross-)correlation spectroscopy, the same procedure was followed but 100 µL of the stock solution of sulfo-Cy5-TTC-U_2-p(DMAc) and Atto425-TTC-0-p(DMAc), respectively, were added to the reaction mixture. The concentration of the V-50 solution was adjusted accordingly to ensure reactant concentrations were comparable to the polymerizations without fluorescent labels.

S1.5. Post-PISA sonication. A 15 mL glass vial containing an aqueous NP dispersion (1 mg/mL, 5 mL) was submerged into an ice bath to prevent overheating. A sonotrode tip (Bandelin MS 73, probe Ø = 3 mm) of an ultrasonic homogenizer (Bandelin GM 2200 combined with a UW 2200 ultrasonic converter, max. power = 200 W) was centrally placed inside the glass vial until 0.5 cm submerged in the liquid. The power delivered by the sonotrode was controlled by an external processor, operating at 50% duty cycle with 10% power (≈ 4 W/mL). The instrument is internally calibrated by the supplier and equipped with an internal signal feedback loop (AMPLICHRON® circuit), which guarantees a constant sonication amplitude and power independent of changing experimental conditions. Samples were sonicated for 30, 60, 120, and 300 s.

S1.6. Centrifugation. Aqueous dispersions of FMs (**P2** – **P5**; at 1 mg/mL) were subjected to the following protocol: 15 min at 500*g* (= **step 1**), 15 min at 2000*g* (= **step 2**), 15 min at 5000*g* (= **step 3**), 30 min at 10000*g* (= **step 4**), 30 min at 15000*g* (= **step 5**), and 30 min at 20000*g* (= **step**

6). All centrifugation steps were done at 20 °C. Aliquots of the supernatant (60 µL) were taken after each centrifugation step and analyzed with DLS, resulting in the *z*-average (of a sphere-equivalent), the PDI, and the derived count rate (± standard deviation for $n = 3$ runs per measurements, respectively). In addition, the samples were measured before and after the centrifugation protocol after gentle redispersing any sedimented particles (= **step 0** and after **step 6**).

S2) Characterization techniques

S2.1. Nuclear Magnetic Resonance (NMR) Spectroscopy. To confirm monomer conversions of DMAc and MEA, ^{1}H-NMR spectroscopy was performed in 5 mm diameter tubes in $CDCl_3$, DMSO-d_6 or acetone-d_6 at 25 °C. Spectra were recorded on an Agilent 400-MR NMR spectrometer (Agilent Technologies) operated at 400 MHz. Residual solvent peaks of $CDCl_3$ (δ = 7.26 ppm), DMSO-d_6 (δ = 2.50 ppm), and acetone-d_6 (δ = 2.05 ppm), respectively, were used to reference the chemical shifts. Data was processed using MestReNova software.

S2.2. Size exclusion chromatography (SEC). Molecular weights M_n (number-average) and M_w (weight-average), as well as the dispersity $Đ = M_w/M_n$ were measured using a Waters 2965 AllianceXC chromatography set up, equipped with a Waters 2414 differential refractive index (dRI) detector (Waters). Separation occurred in two PLgel 5 µm MIXED-D columns (300 × 7.5 mm, linear; Agilent Technologies) in series at 65 °C. DMF with 10 mM LiCl (0.45 µm PTFE filtered) was employed as eluent at a flowrate of 1 mL/min, with an overall runtime of 30 min. Samples were prepared at a typical concentration of 1 – 3 mg/mL and filtered with a 0.2 µm PTFE filter prior to injection. A series of linear polyethylene glycol (PEG) standards of defined and narrow molecular weights (Polymer Standards Service GmbH (PSS)) were used for calibration. Data was recorded and processed with Waters Empower 3 software.

S2.3. Gravimetric determination of the solid content. Aqueous dispersion of TTC-0-p(MEA)-*b*-p(DMAc) or TTC-U_2-p(MEA)-*b*-p(DMAc) or combinations thereof (10 µL) were placed in a tared, open 100 µL aluminum pan. The sample was heated in an oven at 100 °C for 12 h, before weighing the remaining solid. Measurements were performed in triplicates and the results reported as the average value with its standard deviation.

S2.4. Transmission electron microscopy (TEM). Samples for dry-state TEM were prepared by depositing NP dispersions (0.1 wt%, 10 µL) on a 200-mesh Cu-grid (Ted Pella Inc.; Formvar-coated, C-sputtered). After 2 min at RT, excess liquid was removed with blotting filter paper. For negative staining, 5 µL aqueous uranyl acetate solution (2 wt%, 0.22 µm filtered) was added, and excess was removed after 1 min. The samples were left to dry under ambient conditions prior to analysis. TEM was conducted using a Tecnai20 EM operating at 200 kV or a Talos L120C EM operating at 120 kV (Thermo Fisher Scientific).

For cryogenic TEM (cryo-TEM), 3 µL of the aqueous polymer solutions (at 10 mg/mL unless noted otherwise) were deposited on a quantifoil grid. The excess of solution was removed with a Whatman paper, and the grid was immediately frozen in liquid ethane. The observations were carried out at –180 °C by a JEOL JEM-2100 LaB_6 microscope operating at 200 kV. Digital images were recorded on a Gatan Ultrascan 1000 CCD camera.

To estimate the average contour length and thickness of the FMs, particles ($N \geq 100$ particles per sample unless noted otherwise) from dry-state TEM micrographs were manually traced using Adobe Illustrator. FMs that extended beyond the field of view or exhibited substantial overlap with neighboring particles, preventing reliable identification of individual structures, were not included in the analysis. Dry-state TEM rather than cryo-TEM was selected to minimize inaccuracies associated with the lower contrast of cryo-TEM images and the limited number of longer FMs that can be completely imaged within cryo-TEM grid holes. The number-based average contour length (L_n), the weight-based average contour length (L_w), the polydispersity index (L_n/L_w), and the standard deviation (σ) were obtained according to **Eqn. S1 – S3**.[2] The average contour length is reported as $L_n \pm$ standard deviation σ. Equivalent equations were used to determine the number-based average thickness (T_n) and the weight-based average thickness (T_w) of the FMs. The

reproducibility of this approach was assessed by tracing the contour lengths of one image in triplicate as indicated in **Figure S9**. The observed variation between individual analyses of L_n and L_w was considered negligible. Representative contour length distributions throughout this manuscript are depicted as histograms with a bin width of 20 nm.

$$L_n = \frac{\sum_{k=1}^{n} N_k L_k}{\sum_{k=1}^{n} N_k} \quad \textbf{(Eqn. S1)}$$

$$L_w = \frac{\sum_{k=1}^{n} N_k L_k^2}{\sum_{k=1}^{n} N_k L_k} \quad \textbf{(Eqn. S2)}$$

$$\frac{L_w}{L_n} = \left(\frac{\sigma}{L_n}\right)^2 + 1 \quad \textbf{(Eqn. S3)}$$

S2.5. Small angle X-ray scattering (SAXS). SAXS measurements were conducted in transmission mode on a lab-scale instrument, SAXSLAB GANESHA 300XL (Xenocs), equipped with a high brilliance Microfocus Cu Source, Xenocs Genix3D, with wavelength λ = 1.54184 Å. The micellar dispersions and Milli-Q water were loaded into lockable thin-wall quartz capillaries with an inner diameter of 2 mm, and thickness of 0.01 mm. The sample concentration was 1 wt%. The scattered X-rays were collected on a Pilatus 300 K solid-state photon-counting 2D detector at a q range of 0.007 – 0.212 Å^{-1} with an exposure time of 1 h. The scattering vector, q, is related to the scattering angle as indicated in **Eqn. S4**, where λ and 2θ are the wavelength of the incident radiation and the scattering angle, respectively.

$$q = \frac{4\pi}{\lambda} \sin\theta \quad \textbf{(Eqn. S4)}$$

The two-dimensional images obtained from the measurements were averaged azimuthally using SAXSGUI software to obtain the intensity, $I(q)$, vs. q profiles. Prior to the measurements, silver

behenate was used for q-calibration. Milli-Q water was used as a reference to obtain absolute values of the scattering intensities. The SAXS profile of the solvent (Milli-Q water) was subtracted from that of the samples (using ATSAS 3.2.1 (PRIMUS)[3]), and the subtracted profiles are displayed as the average intensity $I(q)$ vs. q. Fitting of the SAXS profiles was performed on the absolute intensity scale (in cm^{-1}). The total measured scattering intensity is expressed as:

$$I(q) = \frac{N}{V}\Delta\rho^2 V^2 P(q) S(q) \quad \textbf{(Eqn. S5)}$$

in which N/V, $\Delta\rho$, V, $P(q)$, and $S(q)$ are the number density, the excess scattering length density (SLD) of the scatterer (compared to that of the solvent), particle volume, form factor, and structure factor, respectively. It was ensured that the samples were measured at sufficiently low concentration to avoid the occurrence of structure factor ($S(q) = 1$). Fitting procedure has been performed in SasView 5.0.6 with the built in model describing a flexible cylinder with elliptical cross section (for detail see SasView documentation).[4,5] In the fitting algorithm the scaling factor, Kuhn-length, cross section equatorial radius and the ellipticity was fitted while the overall length of the particle was kept constant since it is out of range our SAXS setup.

S2.6. Dynamic light scattering (DLS). DLS was carried out with a Malvern Zetasizer Nano S (Malvern Panalytical) to determine the apparent hydrodynamic radius R_h (= z-average diameter / 2) of the particles and their polydispersity index (PDI). Scattered light was detected at 173°. Samples were diluted with Milli-Q water and then measured at 25 °C with $n = 3$, after an equilibration time of 30 s. Data were recorded and processed with Zetasizer Software 8.02.

2.7. Fluorescence (cross-)correlation spectroscopy (F(C)CS). Aqueous, fluorophore containing solutions (100 µL) were loaded into a chambered glass coverslip (#1.5; thickness = 170 ± 5 µm) with 18 individual wells (µ-slide, Ibidi). FCS measurements were performed 30 – 50 µm above the glass surface to prevent surface effects. For each solution, one measurement with a total

duration of 3 min was performed at room temperature (21 °C). FCS measurements were performed on a commercial confocal microscope (Stellaris 8 FALCON FCS, Leica) using a 63×1.2 water immersion objective equipped with motorized correction collar to precisely correct for variations in cover glass thickness (HC PL APO motCORR CS2, Leica). Excitation was achieved by a pulsed white light laser (WLL, $\lambda = 440 – 790$ nm, $\Delta\lambda = 1$ nm, 80 MHz) set at the peak excitation maximum of the fluorophores under investigation (649 nm for sulfo-Cy5 labeled species and 440 nm for Atto425-labeled species). The emitted fluorescence light was collected through the same objective after passing through a confocal pinhole (1 airy unit at 580 nm) and directed to GaAsp-based photon-counting detectors (HyD X) with spectral detection ranges of 660 – 795 and 445 – 585 nm for sulfo-Cy5 and Atto425-labeled species, respectively. The time-dependent fluctuations of the detected fluorescent signal ($F(t)$) were recorded and evaluated by an auto-correlation or cross-correlation function given by the following generalized definition:

$$G_x(\tau) = \frac{\langle F_i(t)F_j(t+\tau)\rangle}{\langle F_i(t)\rangle\langle F_j(t)\rangle} \qquad \textbf{(Eqn. S6)}$$

where τ is the delay time and i and j represent the two orthogonally labeled species. In case $i = j = x$ = sulfo-Cy5 and $i = j = x$ = Atto425, the auto-correlations $G_{\text{sulfo-Cy5}}(\tau)$ and $G_{\text{Atto425}}(\tau)$ are obtained, respectively. The situation i = sulfo-Cy5, j = Atto425, and x = cross yields the cross-correlation $G_{\text{cross}}(\tau)$. The cross-correlations were only qualitatively assessed, making further fitting of the cross-correlation curves not necessary. Conversely, auto-correlation functions were fitted to an analytical expression for n types of freely diffusing fluorescent species through the observation volume (V_{obs}):

$$G(\tau) = \left[1 - T_t + T_t e^{-\frac{\tau}{\tau_T}}\right] \sum_{i=1}^{n} p_i \left(1 + \frac{\tau}{\tau_{d,i}}\right)^{-1} \left(1 + \frac{\tau}{\tau_{d,i}\kappa^2}\right)^{-1/2} + 1 \qquad \textbf{(Eqn. S7)}$$

where T_t represents the fraction of fluorescent labels in the triplet (dark) state, τ_T is the lifetime of the triplet state ($\tau_{T,Atto425} \approx 5$ µs; $\tau_{T,sulfo\text{-}Cy5} \approx 6 - 8$ µs), p_n is the diffusion amplitude (contribution) of the nth diffusing species, $\tau_{d,n}$ is the diffusion time of the nth species and κ corresponds to the structural parameter of the focal volume, which is defined as z_0/ω_0 (≈ 5 – 7 for the used set-up as determined before each measurement session by calibration using a fluorescent probe with a known diffusion coefficient; Fluorescein ≈ 400 µm^2·s^{-1} at 21 °C) with z_0 and r_0 representing the axial and lateral dimensions of V_{obs}. A maximum of $n = 2$ (two components) was used to fit the auto-correlation functions.

The diffusion coefficients of the nth species (D_n) is directly related to their respective diffusion times $\tau_{d,n}$ obtained from the fitting procedure via

$$D_n = \frac{\omega_0^2}{4\tau_{d,n}} \qquad \textbf{(Eqn. S8)}$$

The obtained diffusion coefficients can subsequently be converted to (sphere-equivalent) hydrodynamic radii ($R_{h,n}$) using the Stokes-Einstein relation:

$$R_{h,n} = \frac{k_B T}{6\pi\eta D_n} \qquad \textbf{(Eqn. S9)}$$

where k_B represents the Boltzmann constant, T the temperature and η the viscosity of the surrounding medium (1 mPa·s at 20 °C).

S2.8. Atomic force microscopy (AFM). Prior to FM deposition, 14×14 mm² Si (100) substrates were pre-cleaned by sonication in acetone, ethanol, and deionized water for 10 min each. They were then exposed to UV ozone treatment using a UVO cleaner (Jelight) for 10 min to enhance the Si surface wettability. The FMs, dispersed in aqueous solution (1 mg/mL), were

subsequently deposited onto the substrates by spin-coating at 3600 rpm for 60 s to obtain isolated FMs suitable for AFM imaging.

AFM imaging was performed in PeakForce QNM mode using a Dimension Icon microscope retrofitted with a NanoScope V controller (Bruker, USA) in air at room temperature (~ 21 °C). The AFM data were collected following a sine-wave sample-tip trajectory with a frequency of 2 kHz and a peak-force amplitude value of 30 nm. The ScanAsyst optimization in the user interface was enabled to automatically adjust the feedback loop control. Image processing and data analysis were conducted with the NanoScope (version 9.7) and the NanoScope Analysis software (version 1.7), respectively. Cantilevers (ScanAsyst-Air, Si_3N_4, Al-coated) had a nominal spring constant 0.4 $N{\cdot}m^{-1}$ and silicon-made tips had a nominal radius of 2 nm. Since the AFM measurements are highly sensitive to tip shape, new AFM tips were used in each consecutive experiment. All the AFM images were captured at 512×512 pixels.

S2.9. Statistical reporting. For all experimentally determined parameters and values, reported variations are expressed as standard deviations.

S3) Polymer synthesis and characterization

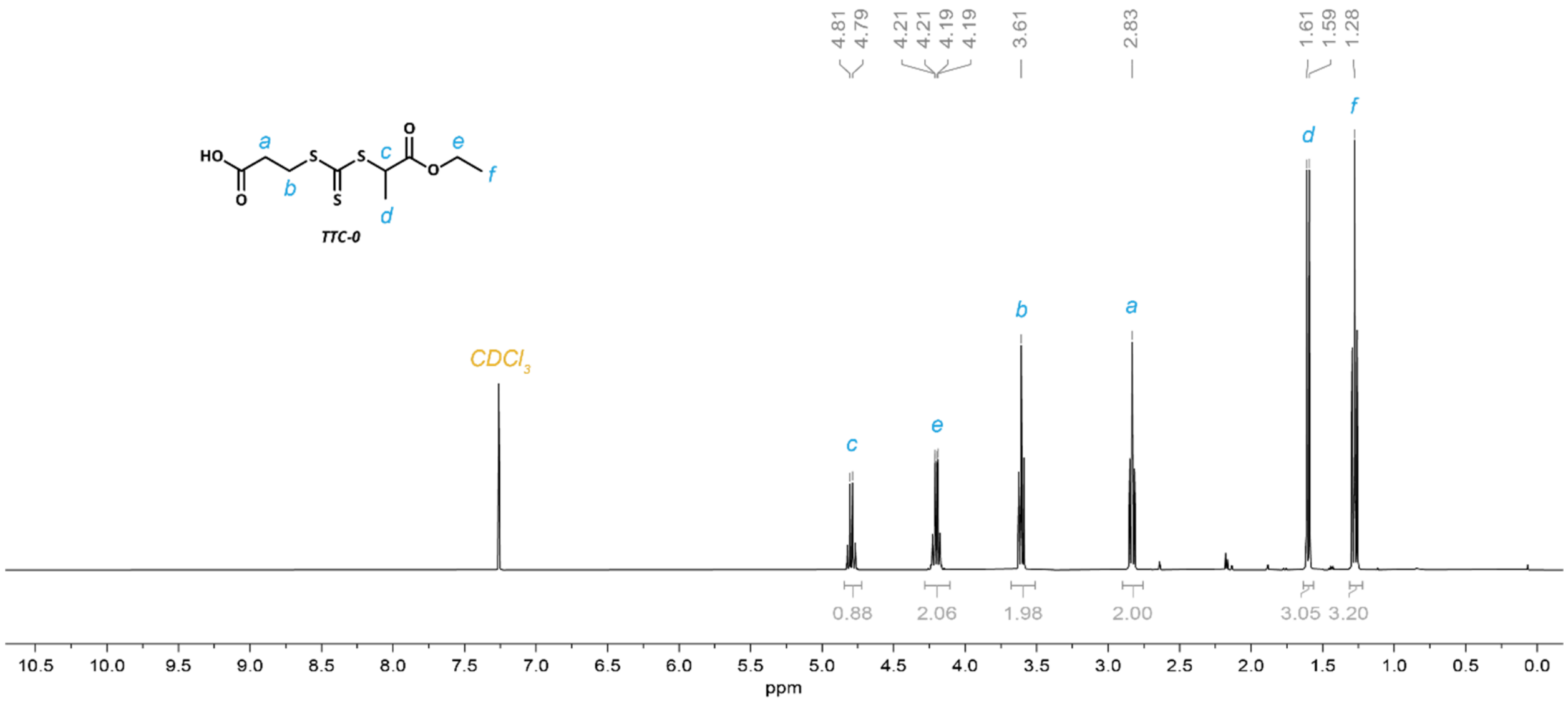

Figure S1. ^{1}H-NMR spectrum of TTC-0, recorded in $CDCl_3$.

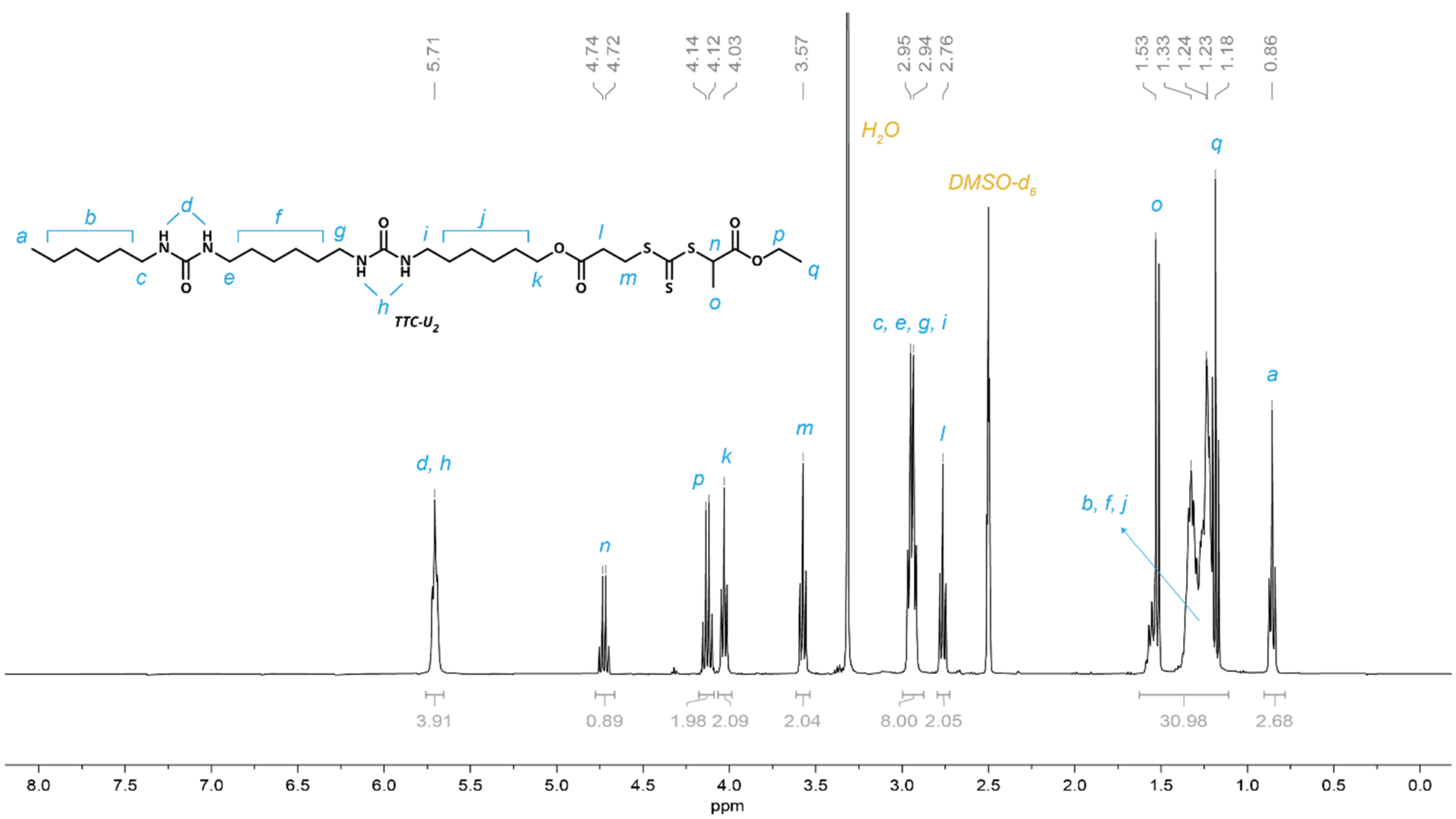

Figure S2. ^{1}H-NMR spectrum of TTC-U_2, recorded in DMSO-d_6.

Table S1. Experimental conditions and results for macroCTA synthesis via RAFT polymerization of DMAc in the presence of TTC-U_2 (**M1**) or TTC-0 (**M2**). $[DMAc]_0/[TTC]_0 = 42$, $[DMAc]_0 = 2.2$ M.

Expt	TTC	$[TTC]_0$/ $[AIBN]_0$	X (%) (time in min)	DP_{NMR}	$M_{n,th}$ (kg/mol)	$M_{n,SEC}$ (kg/mol)	$M_{w,SEC}$ (kg/mol)	$Đ$ (-)
**M1**	TTC-U_2	20.8	77 (75)	33	3.9	2.5	2.9	1.15
**M2**	TTC-0	20.5	69 (75)	29	3.2	-	-	-

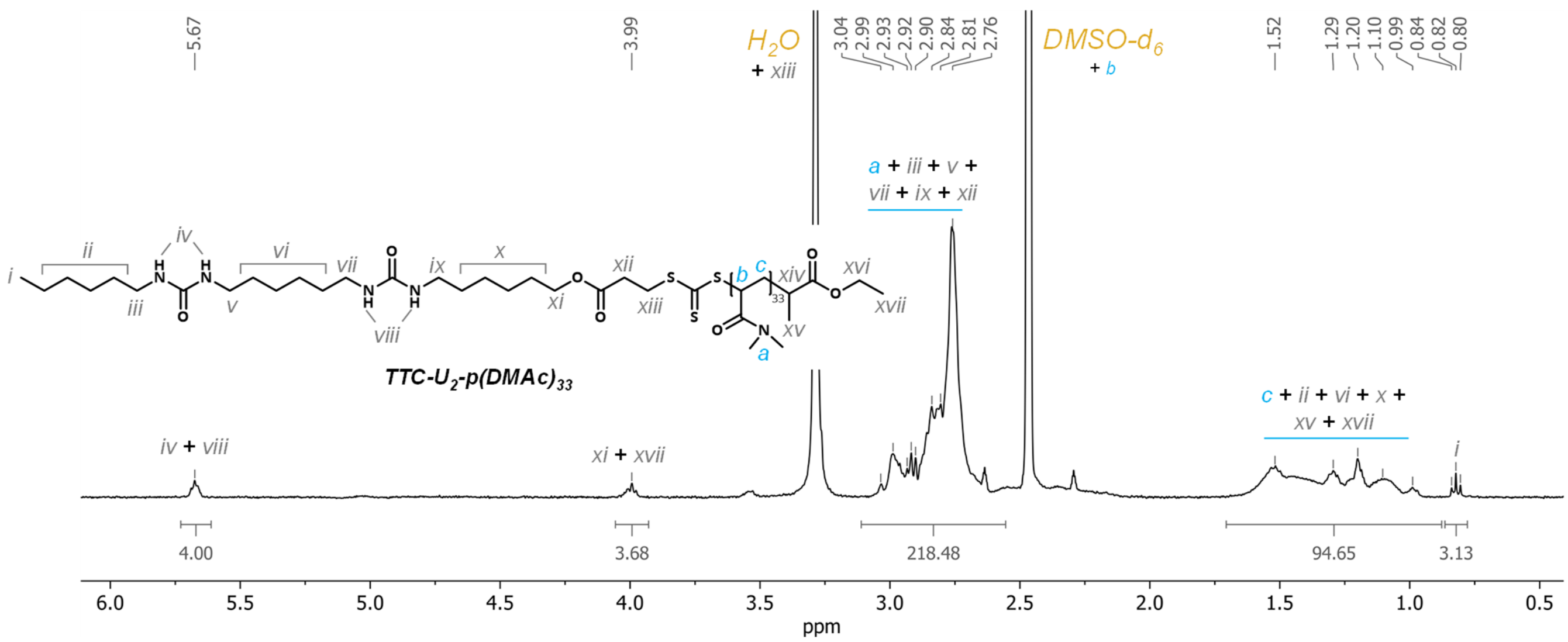

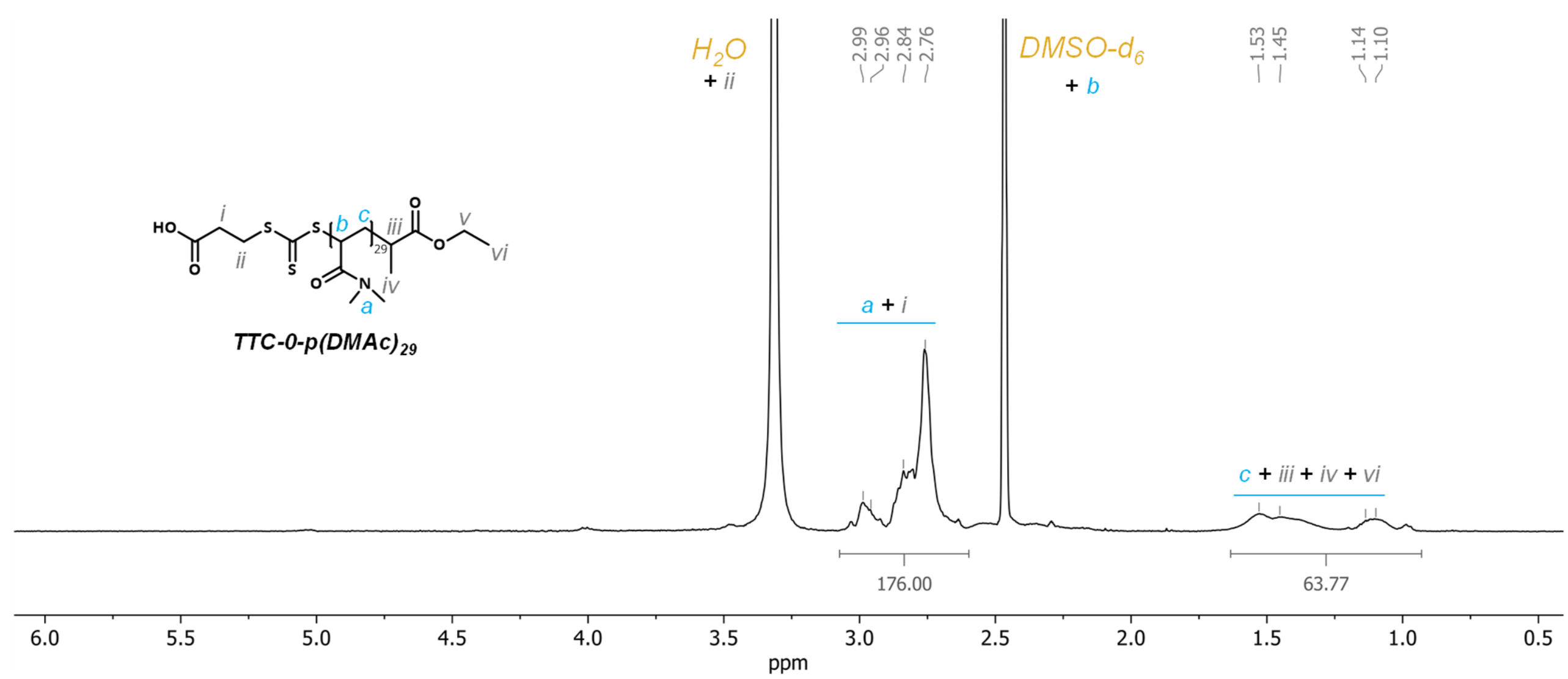

Figure S3. ^{1}H-NMR spectra of the macroCTAs TTC-U_2-p(DMAc)$_{33}$ (top) and TTC-0-p(DMAc)$_{29}$ (bottom), recorded in DMSO-d_6. Note that not all signals could be identified due to overlapping solvent and/or polymer signals.

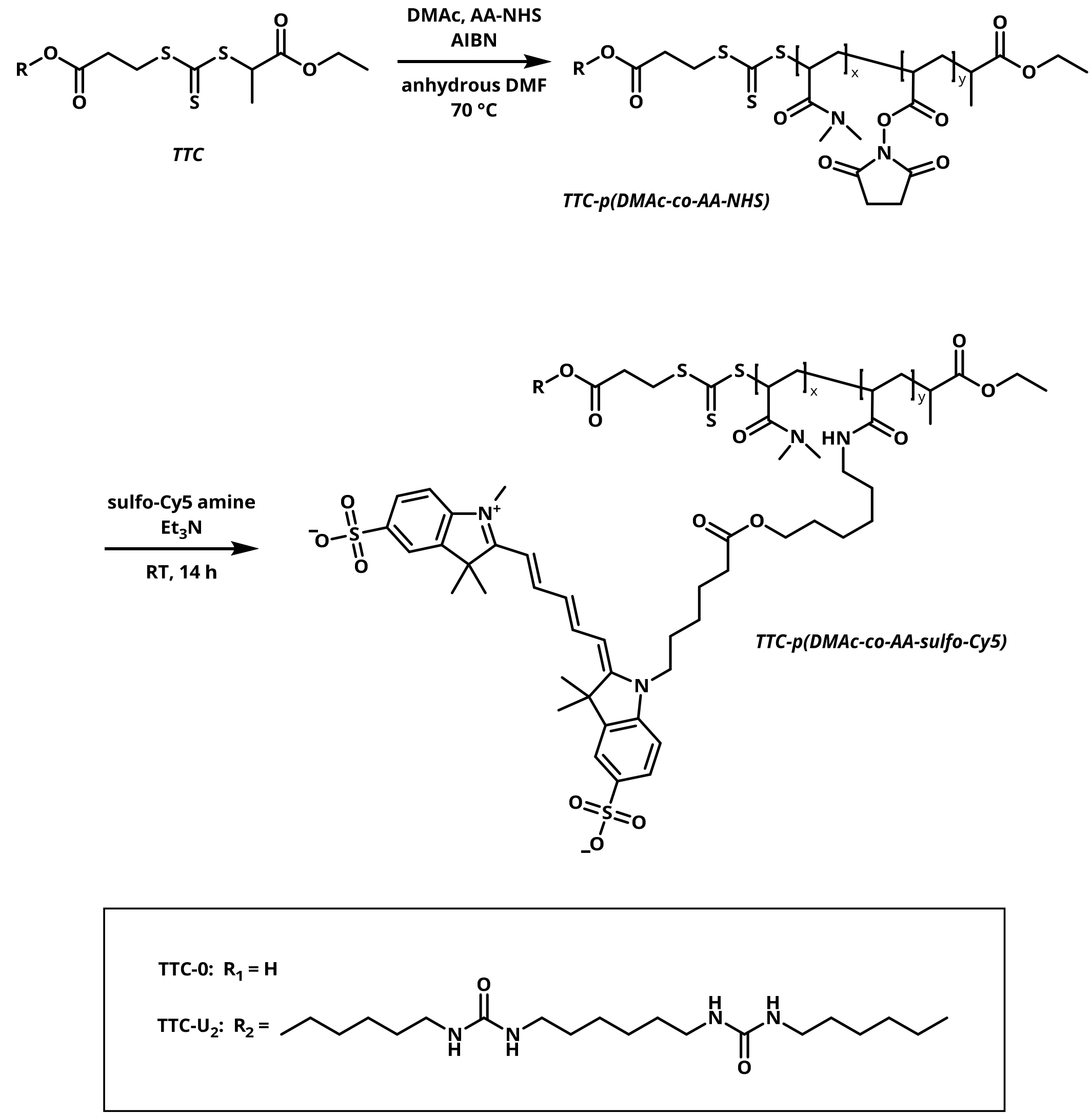

Figure S4. Fluorescent labelling of TTC-p(DMAc) macroRAFT agent with sulfo-cyanine5 amine. The same strategy was applied for labelling with Atto425 amine.

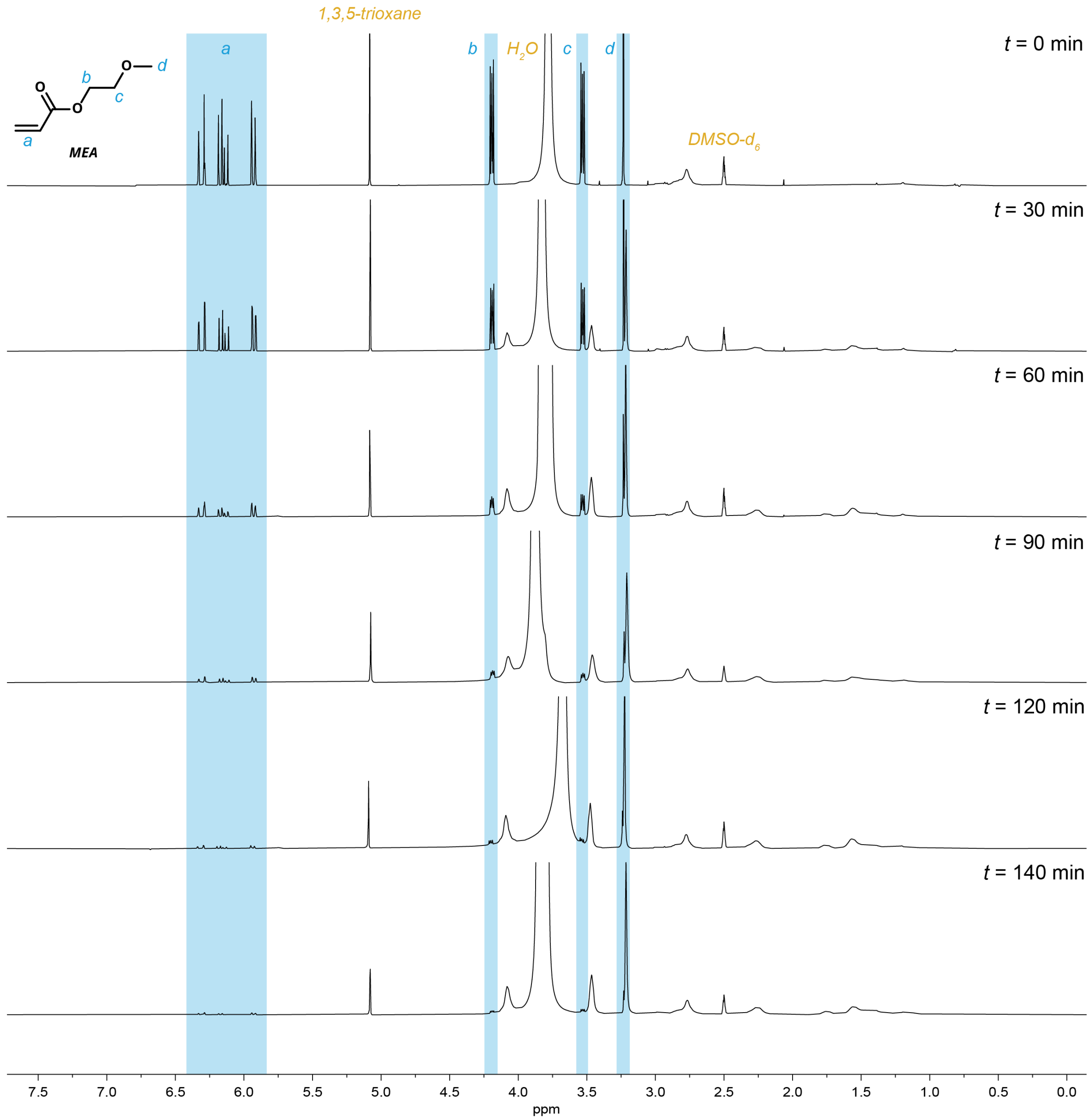

Figure S5. ^{1}H-NMR spectra of kinetic samples taken during the RAFT dispersion polymerization of MEA with TTC-U$_2$-p(DMAc)$_{33}$. MEA conversion was determined by comparing the vinyl peak of MEA (5.9 ppm) to the internal standard 1,3,5-trioxane (5.1 ppm). Spectra were recorded in DMSO-d_6.

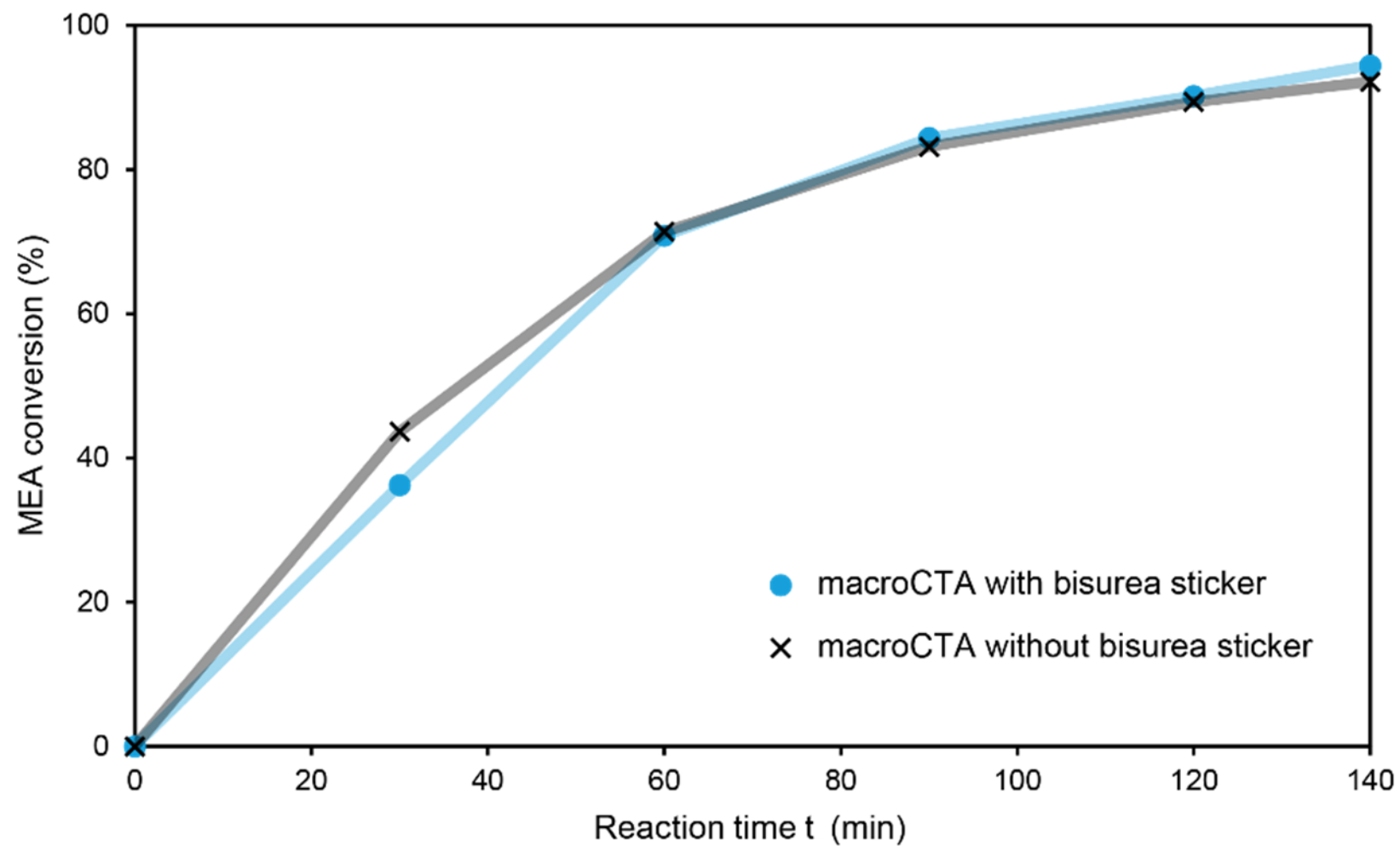

Figure S6. Conversion of MEA during block copolymer synthesis via aqueous PISA with TTC-U_2-p(DMAc) (macroCTA with bisurea sticker) and TTC-0-p(DMAc) (macroCTA without bisurea sticker), respectively. The MEA conversion was determined via ^{1}H-NMR as outlined in **Figure S5**.

Table S2. Aqueous RAFT-PISA (**P**) with MEA at 60 °C synthesis overview. $[MEA]_0/[TTC\text{-}p(DMAc)]_0 = 99$, $[MEA]_{0,aq} = 0.86$ M, $[TTC]_0/[AIBN]_0 = 10$.

Expt.	macroCTA ratio TTC-0:TTC-U_2	X_{MEA} [a] (%)	$DP_{MEA,NMR}$ [b] (-)	$M_{n,th}$ [b] (kg/mol)	$M_{n,SEC}$ [c] (kg/mol)	$M_{w,SEC}$ [c] (kg/mol)	$Đ$ [c] (-)	Morphology [d]
**P7**	25:75	92	91	15.2	9.6	11.7	1.22	Fibers
**P8**	35:65	96	95	16.0	10.2	13.5	1.32	Fibers
**P9**	45:55	85	84	14.2	3.7	5.1	1.31	Fibers
**P10**	60:40	88	87	14.5	4.1	5.4	1.39	Fibers

[a] Monomer conversion (X_{MEA}) was determined at the end of the reaction (t = 140 min) by ^{1}H-NMR, using 1,3,5-trioxane as internal standard. [b] Theoretical degree of polymerization (DP) and number-average molar mass, M_n, based on the experimentally determined MEA conversion. For macroCTA combinations the weighted average is reported. [c] Number-average molar mass, M_n, weight-average molar mass, M_w, and dispersity index, $Đ = M_w/M_n$, determined by SEC in DMF (+ 10 mM LiCl) using PEG standards for calibration. [d] Determined by TEM of aqueous NP dispersions (1 mg/mL) after staining with 2% aqueous uranyl acetate solution.

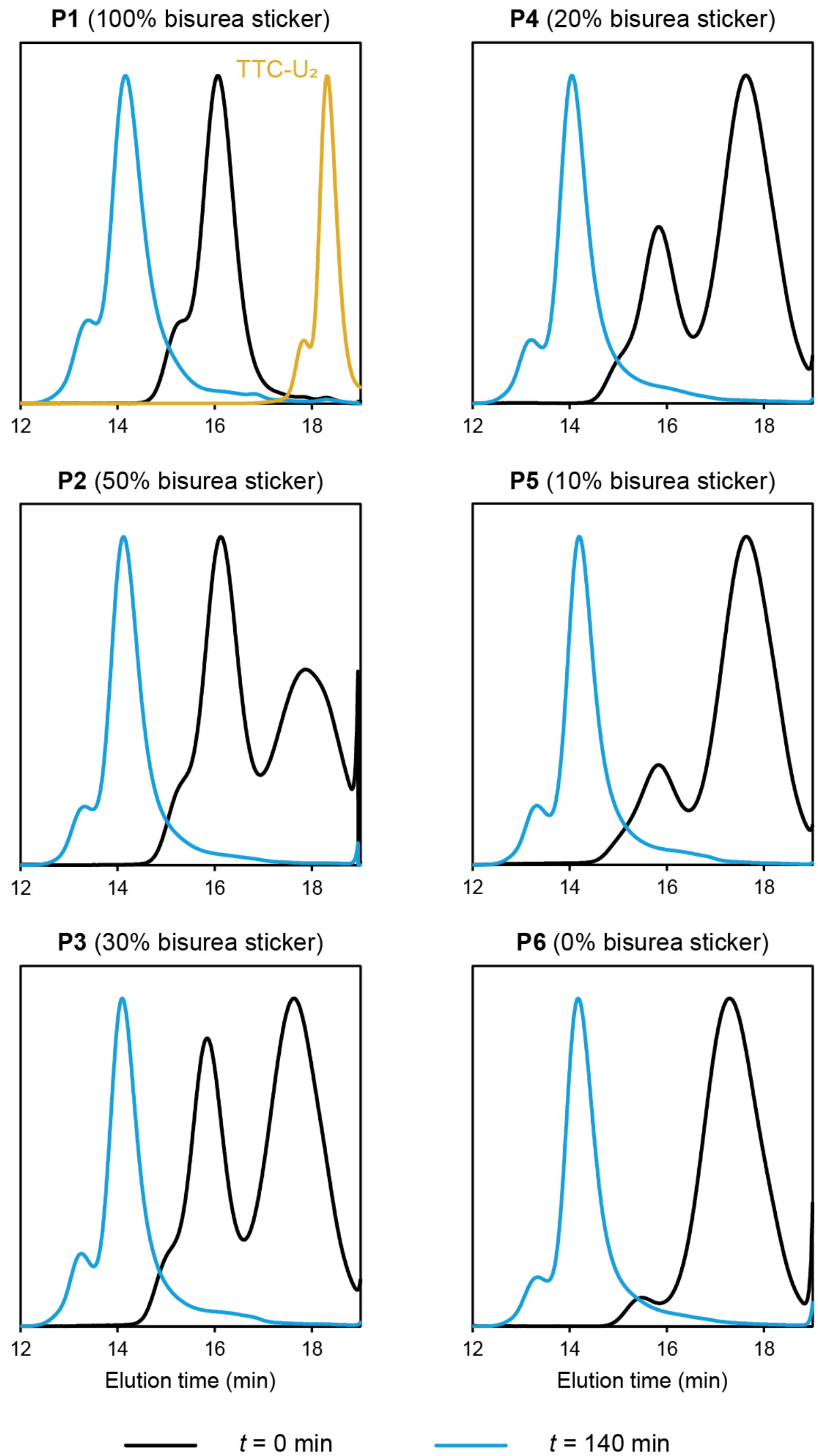

Figure S7. SEC traces (dRI) of TTC-0/U_2-p(MEA)$_y$-*b*-p(DMAc)$_x$ (**P1 – P6**) at $t = 0$ min (black lines, indicative of the respective macroCTAs) and $t = 140$ min = end of the reaction (blue lines). Subsequent purification with dialysis did not lead to any changes of the SEC traces of the BCPs. For **P1** (100% bisurea sticker content), also the SEC trace of TTC-U_2 is shown in yellow. The trace of TTC-0 is not shown as insufficient separation from the solvent peak occurred due to its low molecular weight.

S4) TEM image analysis

S4.1. Schematic representation and TEM verification of cut and capped FM ends

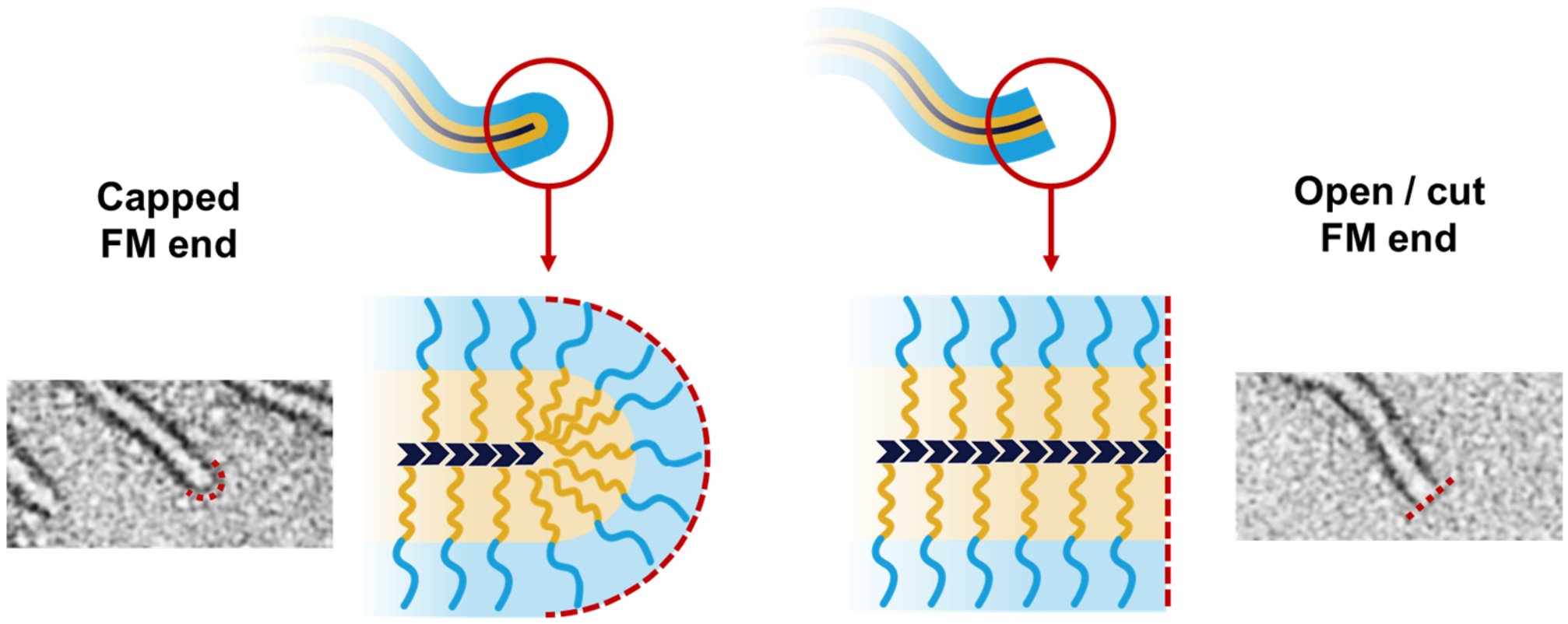

Figure S8. A schematic representation of filomicelles (FMs) with (left) capped and (right) cut/open ends together with representative examples of both FM ends as observed in TEM images.

S4.2. Dry-state TEM of FMs: Average contour length + width & robustness of tracing

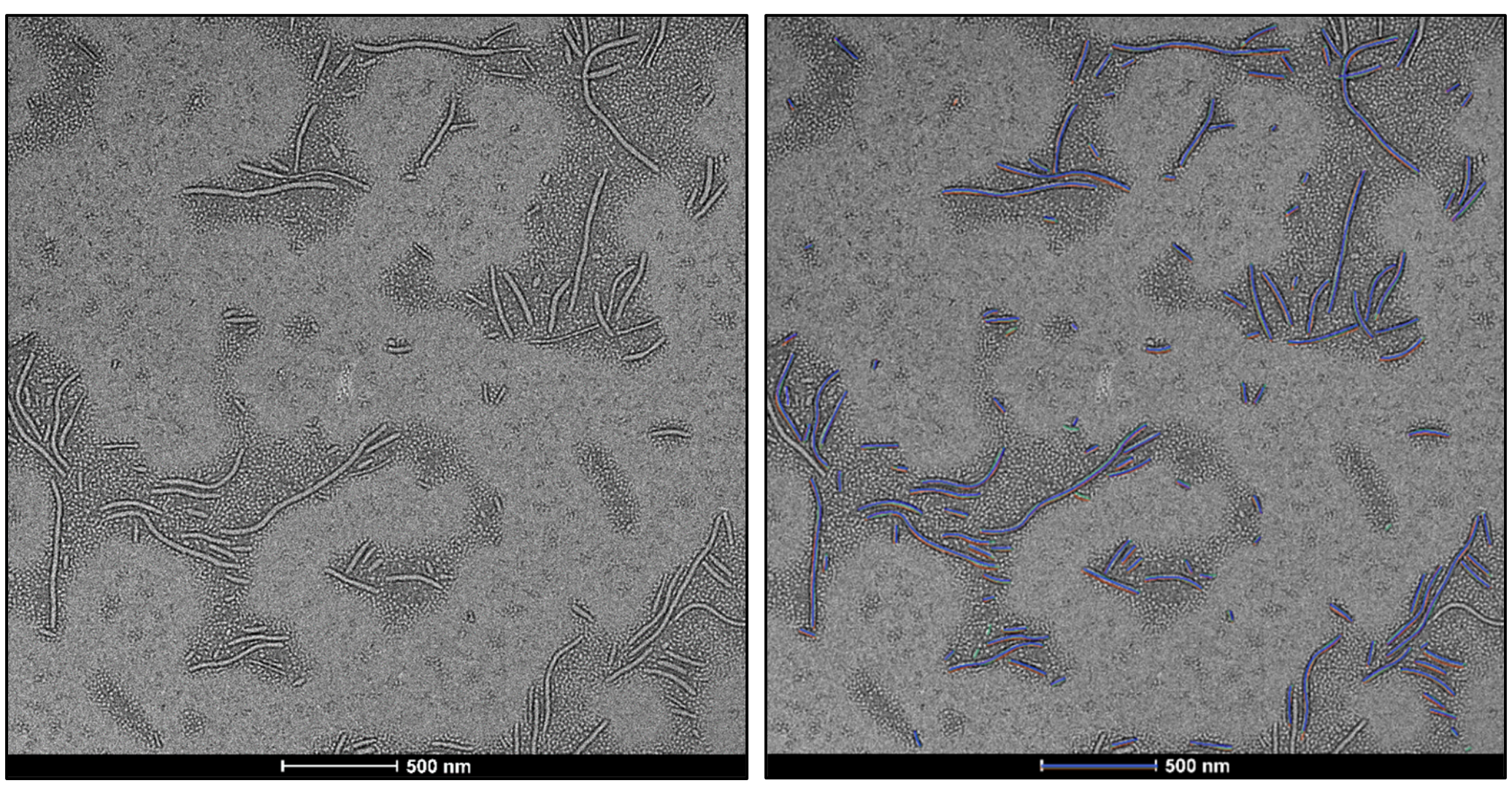

	Particle count N	L_n (nm)	L_w (nm)	PDI = L_w/L_n
**1**	121	185.9	356.5	1.92
**2**	124	185.2	362.5	1.96
**3**	123	185.4	360.6	1.95
**Average**	123 ± 1.5	185.5 ± 0.4	359.9 ± 3.1	1.94 ± 0.02

Figure S9. A representative TEM micrograph of filomicelles (FM, left) was traced in triplicate (overlay on the right) to estimate the manual tracing error during image analysis. Error given is ± standard deviation. The unidentified features surrounding the FMs are believed to be either impurities from the staining solution used to prepare the TEM samples or inconsistencies in the TEM grid surface which preferentially attracts the FMs during the drying process.

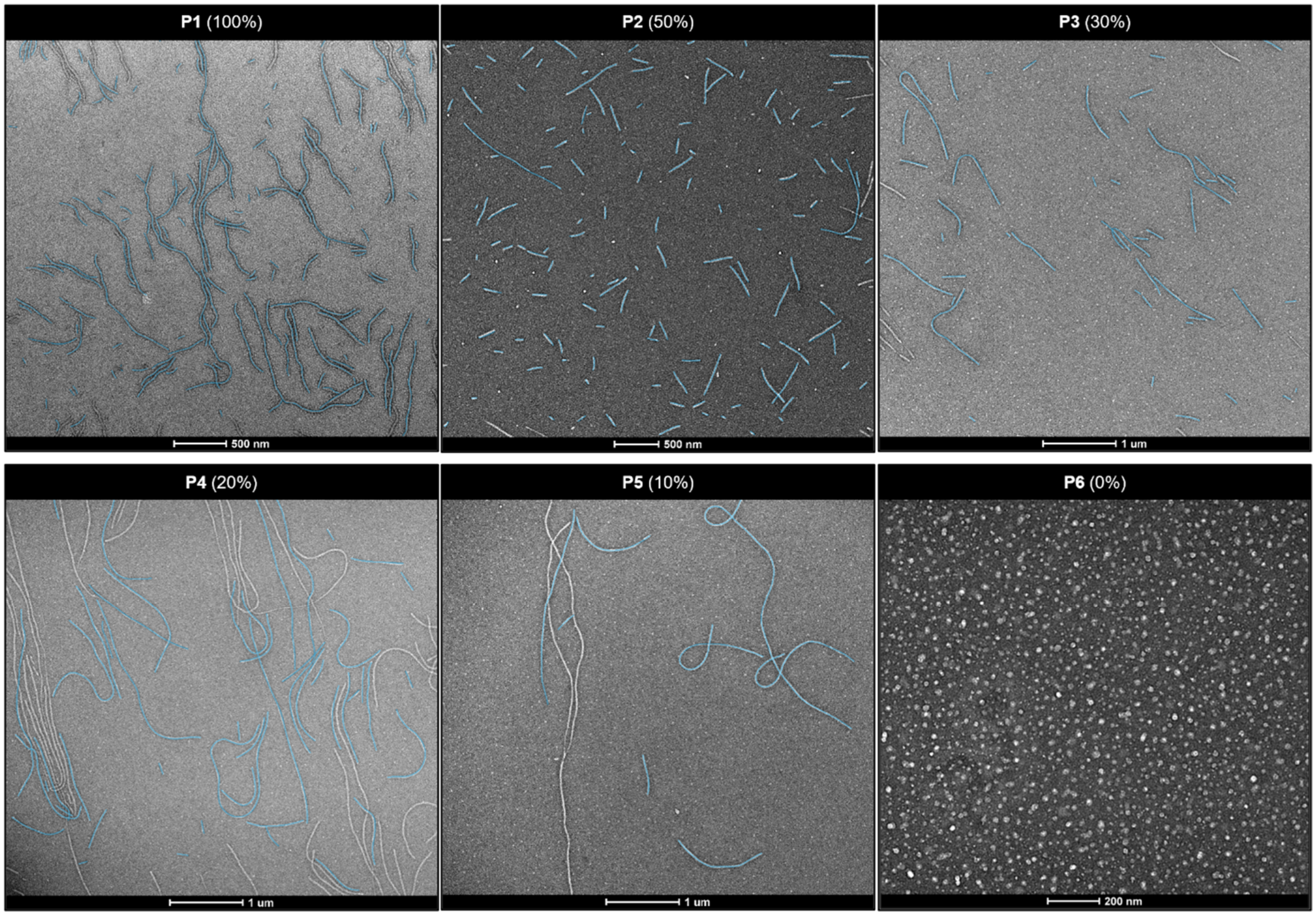

Figure S10. TEM micrographs of **P1 – P6**, complementary to zoomed images shown in **Figure 3C**.

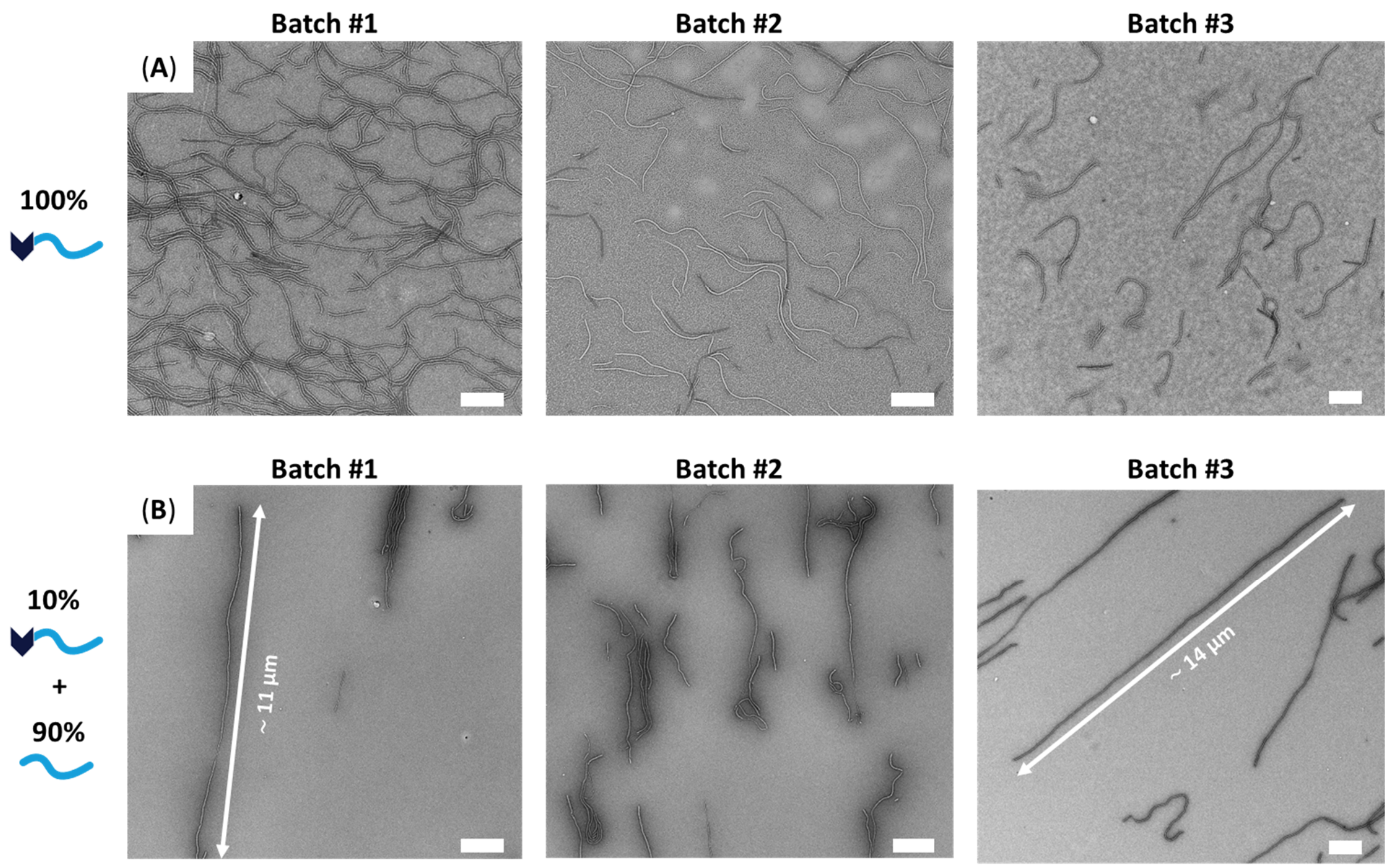

Figure S11. Reproducibility of filomicellar morphology of (**A**) **P1** (TTC-0: TTC-U_2 = 0:100) and (**B**) **P5** (TTC-0: TTC-U_2 = 90:10) as probed with TEM on three independently synthesized particle batches. Scale bar = 500 nm for panel **A** and 1 µm for panel **B**. Filomiclles with lengths up to approximately 14 µm were observed for **P5**.

S4.3. Determination of the persistence length *P* with Easyworm[6]

Easyworm is an open source, MATLAB-based software tool developed by Lamour et al. that facilitates analyzing shape fluctuations of worm-like chains and allows for accurate measurements of their persistence length *P*. Worm-like chains are first manually traced on microscopy images to fit their contour to a parametric spline. Within Easyworm, *P* can then be calculated via three distinct measures which are derived from the worm-like chain model (WLC) for semi-flexible polymers. This software tool is able to provide reliable results over a wide range of *P* (ranging from that of DNA, $P \approx 50$ nm, to that of microtubules, $P \approx 5.2$ mm), making it suitable for the filomicelles (FMs) described in this work: Given their average contour lengths L_n of 0.2 – 1.9 µm (see **Figure 3** and **5**), the method to determine *P* based on the mean square of the end-to-end distances *R* was selected (see **Figure S12** and **Eqn. S10**).

$$\langle R^2 \rangle = 2sPl\left(1 - \frac{sP}{l}\left(1 - e^{-\frac{l}{sP}}\right)\right) \quad \textbf{(Eqn. S10)}$$

Beyond the manually traced TEM micrographs (see **Figure S9** and **S10** for representative images), the following input parameters were selected for the data analysis: The samples for dry-state TEM were immobilized at room temperature, hence the temperature in 'Easyworm 2' was set to 21 °C. The same TEM images that were used to determine the contour length L_n previously were used here. Since equilibration on the surface could be guaranteed, the surface parameter *s* was set to 1.5 ± 0.5 (corresponds to non-equilibrium fit).

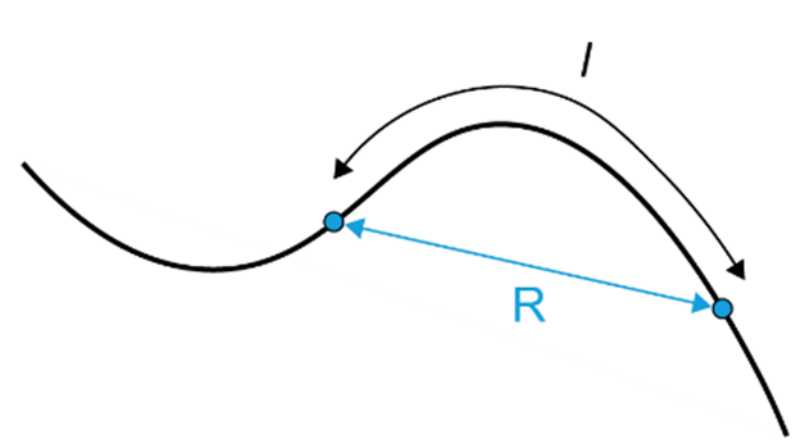

Figure S12. Strategy used to determine the persistence length *P* based on the mean square of end-to-end distances *R* as a function of *l* according to **Eqn. S4**. *s* is a surface parameter set to 1.5 ± 0.5 for unequilibrated chains. Scheme was adjusted from Lamour et al.[6]

Table S3. Determination of the persistence lengths P and contour lengths $L_{n,EW}$ with Easyworm (= EW) and comparison to previously determined contour lengths L_n. N is the number of counted particles. For completeness, the average thickness (T_n and T_w) and aspect ratios (L_w/T_w or L_w/T_{SAXS}) are also included.

Sample overview		Manual tracing						Easyworm				
**Sample**	**MacroCTA ratio TTC-0:TTC-U$_2$**	N **(-)**	L_n **(L_w) (nm)**	L_w/L_n **(-)**	T_n [a] **(T_w) (nm)**	L_w/T_w **(-)**	L_w/T_{SAXS} [b] **(-)**	N_{EW} **(-)**	$L_{n,EW}$ [c] **(nm)**	**Sum contour lengths (µm)**	P [d] **(nm)**	$L_{n,EW}/P$ **(-)**
**P1**	0:100	170	305 (677)	2.2	33.7 (33.9)	20	9	99	552 ± 612	54.7	1494 ± 682	0.37
**P1** (cryo-TEM[e])	0:100	-	-	-	-	-	-	36	479 ± 399	17.3	533 ± 234	0.90
**P2**	50:50	253	241 (494)	2.1	32.6 (33.3)	15	7	73	408 ± 408	29.8	962 ± 348	0.42
**P3**	70:30	231	400 (724)	1.8	31.8 (34.2)	21	11	64	550 ± 436	35.2	392 ± 311	1.4
**P4**	80:20	214	1008 (1999)	2.0	29.5 (30.1)	67	28	67	1173 ± 1249	78.6	468 ± 317	2.5
**P5**	90:10	83	1913 (3460)	1.8	30.9 (32.1)	108	54	30	2613 ± 1551	78.4	852 ± 450	3.1

[a] Determined from dry-state TEM images on 30 individual particles. [b] T_{SAXS} = 35.5 nm. To account for the ellipticity of the cross-section, T_{SAXS} was obtained by taking the average of the short (= 2 · 14.5 = 29 nm) and long axis (= 2 · 1.45 · 14.5 = 42 nm) extracted form SAXS form factor fitting. The factor of 1.45 represents the average ellipticity. [c] Contour lengths $L_{n,EW}$ are reported as number-based average ± standard deviation. [d] P (Contour/End-to-end) was determined from the measurement of the mean square of the end-to-end distances R as a function of l (suitable for semiflexible worm-like chains). [e] See **Figure S13**.

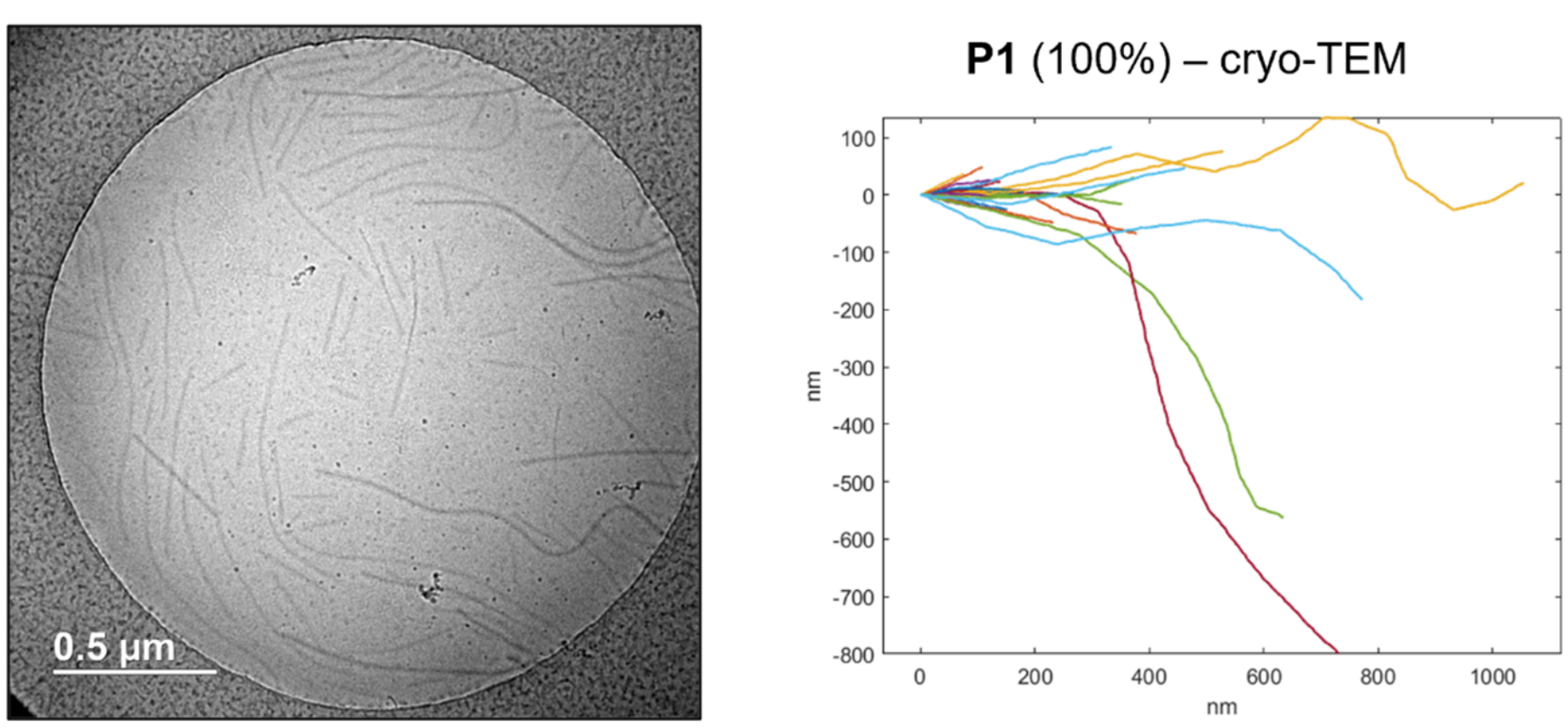

Figure S13. Cryo-TEM micrograph of **P1** (left) considered for image analysis with Easyworm to determine the persistence length P. On the right, the corresponding chains (smoothed traces) with initial tangents aligned.

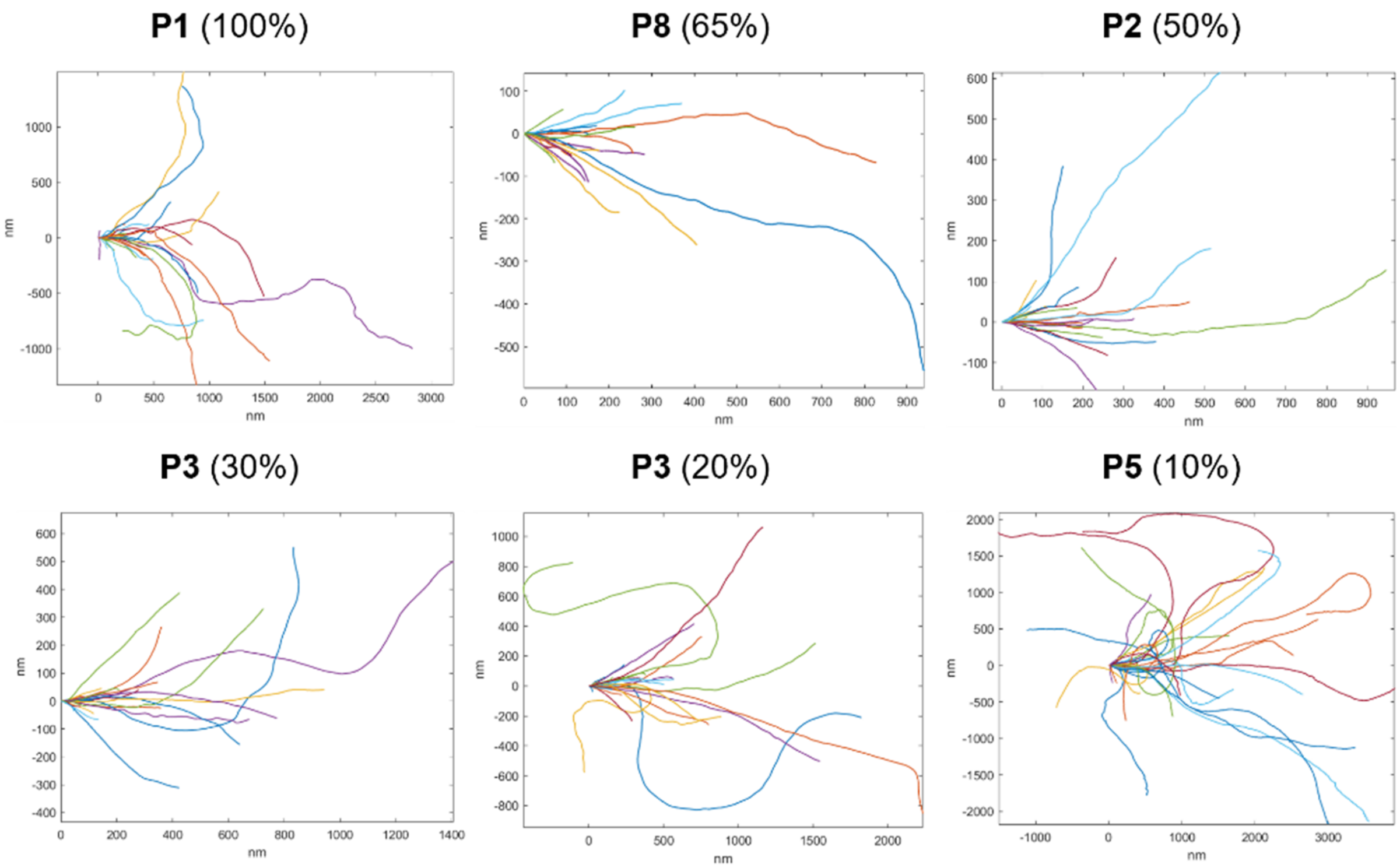

Figure S14. Chains (smoothed traces, randomized) with initial tangents aligned for samples **P1**-**P5** and **P8**.

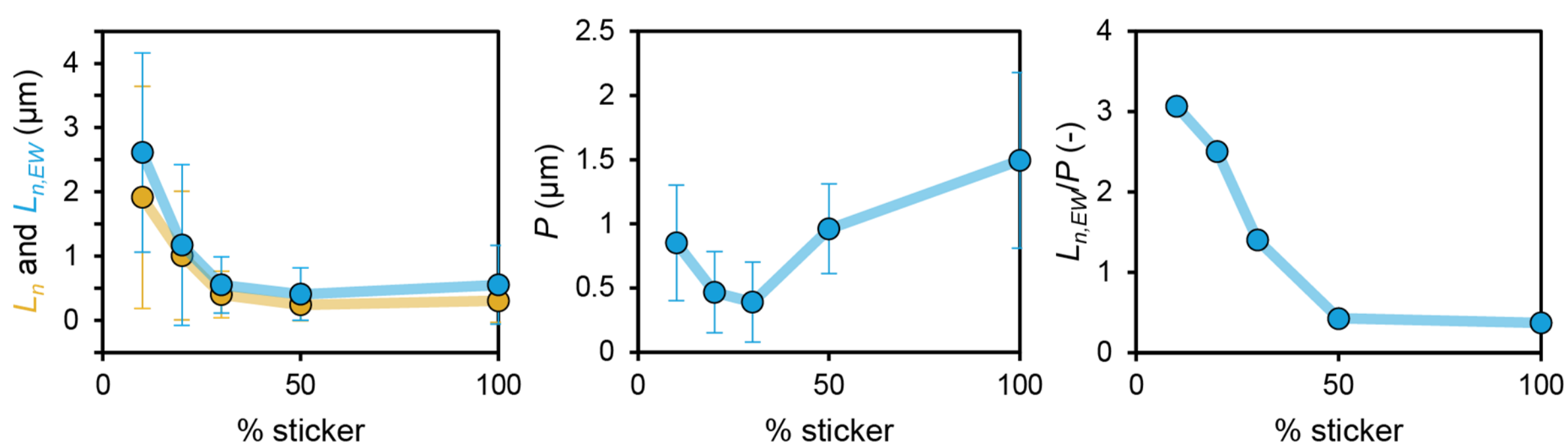

Figure S15. Results from Easyworm analysis of **P1** – **P5** as a function of the bisurea sticker content (displayed data corresponds to **Table S3**): (**A**) Average contour length (± standard deviation) after manually tracing/counting with Adobe Illustrator (L_n; yellow markers) and with Easyworm ($L_{n,EW}$; blue markers). (**B**) Persistence length P. (**C**) Ratio of the contour length and the persistence length ($L_{n,EW}/P$).

S5) Overlap concentration (c^*) estimation of TTC-0-p(DMAc)

The overlap concentration (c^*) of TTC-0-p(DMAc) can be estimated according to[7]:

$$c^* = \frac{M}{\frac{4}{3}\pi R_g^3} \quad \textbf{(Eqn. S11)}$$

with M the polymer molecular weight (= 3000 g/mol; DP ≈ 30) and R_g the radius of gyration. R_g was extracted from combing the Flory-Fox equation (**Eqn. S12a**)[8–10] with a reported relation of the intrinsic viscosity ($[\eta]$ in $cm^3 \cdot g^{-1}$) and the molecular weight of poly(DMAc) homopolymers dissolved in water at 25 °C (**Eqn. S12b**)[11]:

$$[\eta] = 6^{3/2}\Phi \frac{R_g^3}{M} \quad \textbf{(Eqn. S12a)}$$

$$[\eta] = (9.26 \cdot 10^{-2}) \cdot M^{1/2} \quad \textbf{(Eqn. S12b)}$$

$$R_g = \left(\frac{9.26 \cdot 10^{-2} M^{3/2}}{6^{2/3}\Phi}\right)^{1/3} \quad \textbf{(Eqn. S12c)}$$

Given that the Flory's viscosity factor (Φ) ≈ $2.5 \cdot 10^{23}$ mol^{-1}, a R_g ≈ 1.6 nm is found (*Note*: this value is consistent with experimentally determined hydrodynamic radii measured with fluorescence correlation spectroscopy; see **Table S4**, despite ignoring the CTA chain-end on these relatively short polymers). Plugging the obtained R_g into **Eqn. 11** yields a theoretical c^* of 48 mg/mL.

Based on the quantities listed in **Section S1.4.** of the **Materials & methods**, the total macroCTA concentration at the start of a *t*-PISA reaction is approximately 24 mg/mL and therefore roughly 2× lower than the theoretical c^*.

S6) Characterization of macroCTA preassemblies with DLS and F(C)CS

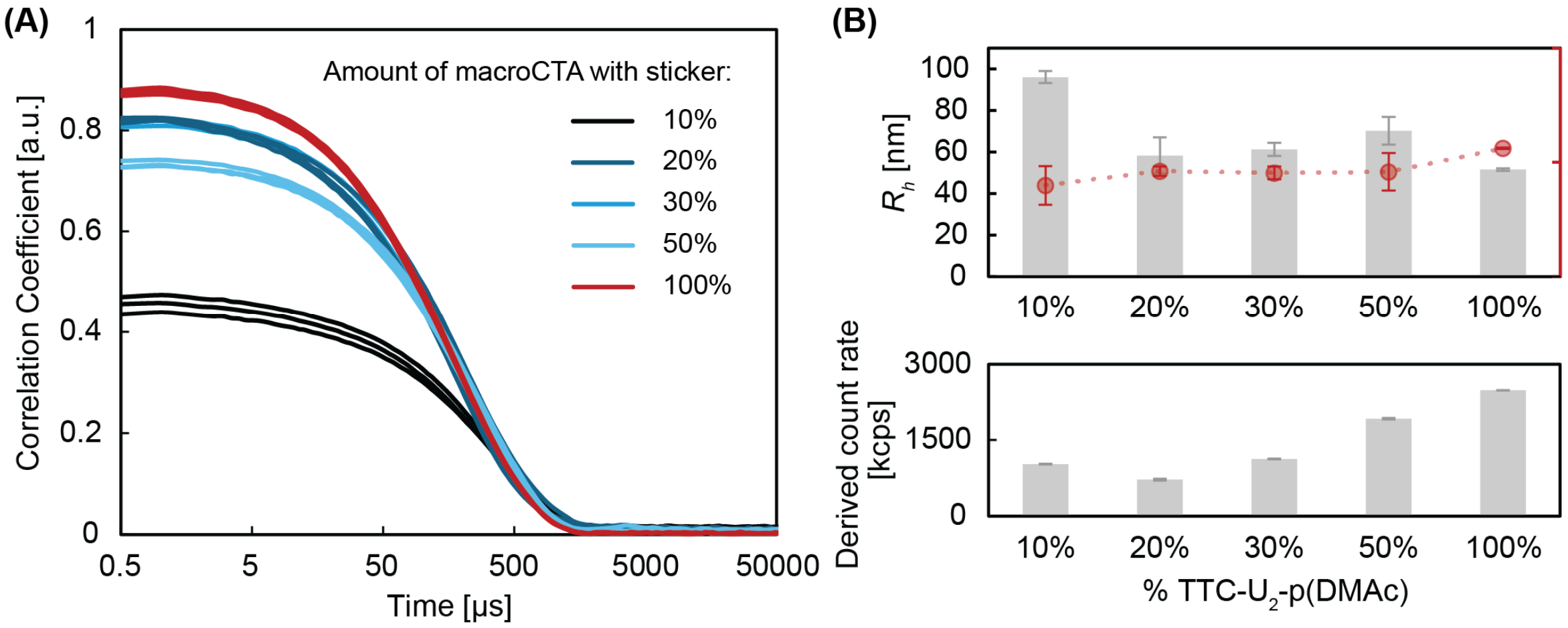

Figure S16. DLS of combinations of macroCTAs with and without sticker at different molar ratios in water (sample concentration: 0.6 mg/mL): (**A**) Correlation curves and (**B**) Hydrodynamic radius of a sphere-equivalent (R_h, in nm) and polydispersity (PDI) on the top and the derived count rate (in kcps) on the bottom. Reported errors are ± standard deviation (n = 3 measurements).

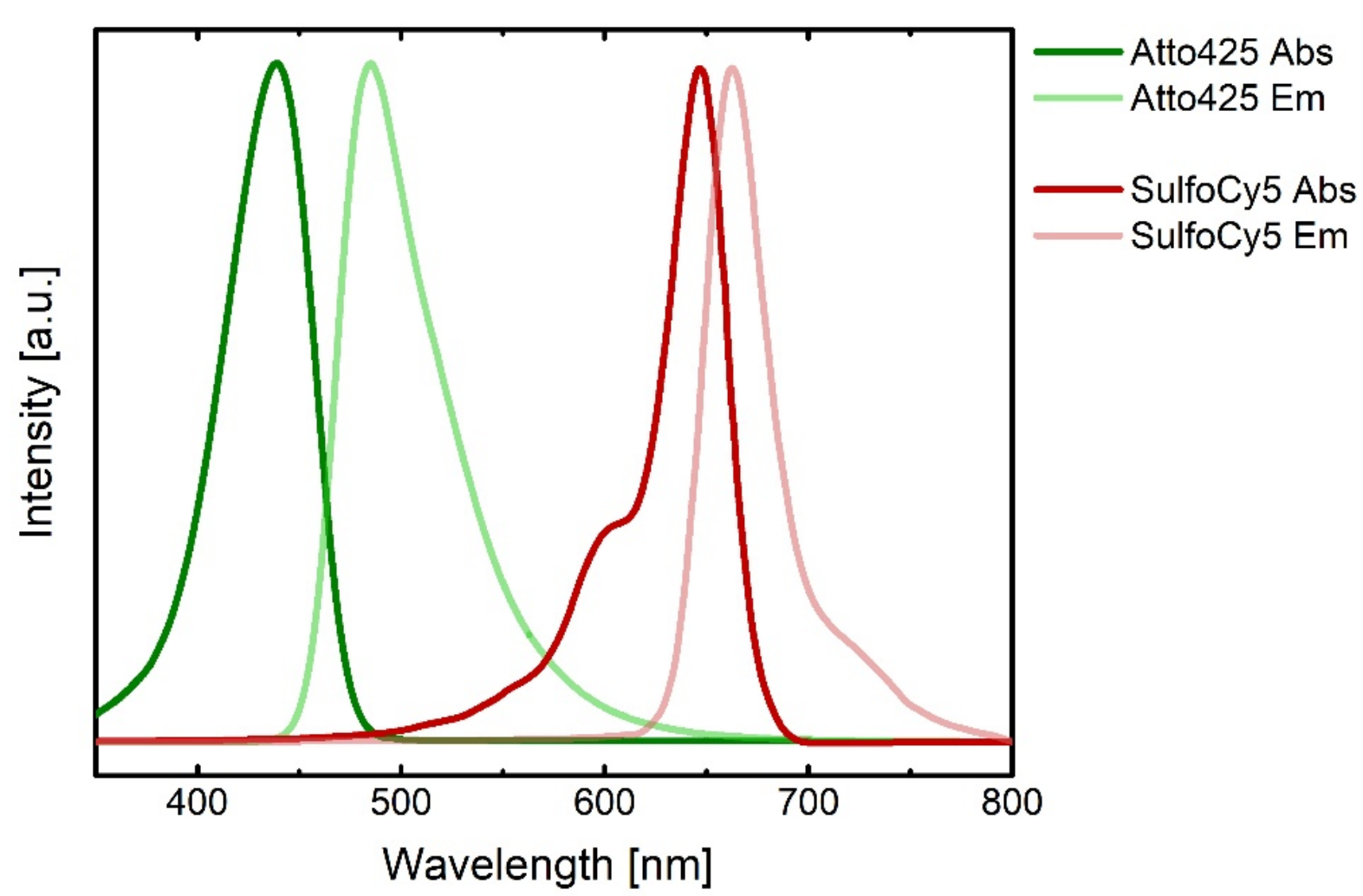

Figure S17. Absorption (darker colors) and fluorescence emission spectra (lighter colors) of Atto425 (green) and Sulfo-Cy5 (red).

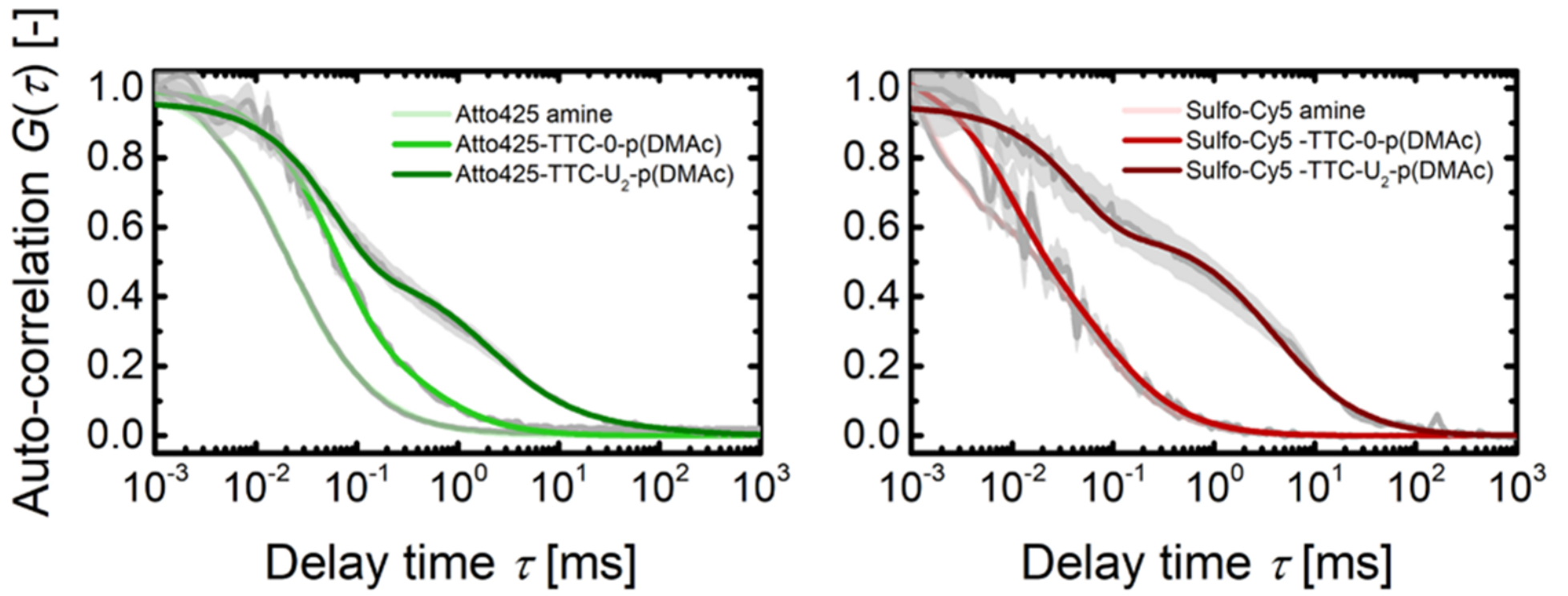

Figure S18. Left: Auto-correlation curves of Atto425-labelled macroCTAs with (TTC-U_2-p(DMAc), dark green) and without (TTC-0-p(DMAc), bright green) the bis-urea sticker. Freely dissolved Atto425 amine is included as a reference (light green). Right: Same as in the left panel, but with sulfo-Cy5 labelling. The measured auto-correlation functions are plotted in dark grey with the lighter grey area indicating the experimental error. The colored lines are fits based on **Eqn. S7**. For Atto425-TTC-U_2-p(DMAc) and Sulfo-Cy5-TTC-U_2-p(DMAc) a 2-component fit was applied, while all other auto-correlation functions could be fitted with a 1-component model. The extracted diffusion coefficients (D) are reported in **Table S4**.

Table S4. FCS results of individual fluorescent macroCTA species as well as the respective small molecule fluorescent dyes for reference. Hydrodynamic radii R_h were determined from D via the Stokes-Einstein equation (**Eqn. S9**).

**Fluorescent species**	$\tau_{Triplet}$ [a]		***D*** **(µm²/s)**	R_h **(nm)**
**Atto425 amine**	5 µs	fitted	460 ± 22	0.47
**Atto425-TTC-0-p(DMAc)**	5 µs	fixed	150 ± 20	1.43
**Atto425-TTC-U$_2$-p(DMAc)**	5 µs	fixed	D_1 = 151 ± 0<br>D_2 = 1.04 ± 0.06	$R_{h,1}$ = 1.42<br>$R_{h,2}$ = 206
**Sulfo-Cy5 amine**	1.56 µs	fitted	204 ± 4.3	1.05
**Sulfo-Cy5-TTC-0-p(DMAc)**	8 µs	fixed	132 ± 16	1.63
**Sulfo-Cy5-TTC-U$_2$-p(DMAc)**	8 µs	fixed	D_1 = 133 ± 46<br>D_2 = 1.54 ± 0.09	$R_{h,1}$ = 1.61<br>$R_{h,2}$ = 139

[a] For Atto-labeled species: The triplet life-time ($\tau_{Triplet}$) of 5 µs was determined from fitting the auto-correlation functions obtained from solutions containing free Atto425 amine dye. For subsequent fits, ($\tau_{Triplet}$) was fixed at this value. For Sulfo-Cy5-labeled species: A $\tau_{Triplet}$ of 1.56 µs was found for the free dye. However, for polymeric sulfo-Cy5 labeled species, the auto-correlation functions could not be fitted accurately. The auto-correlation data could best be described with $\tau_{Triplet}$ = 8 µs. Variations in triplet life-time upon dye conjugation are common and can be caused by differences in local environments. The obtained value 8 µs stays below the threshold of 10 µs for typical organic fluorescent labels.

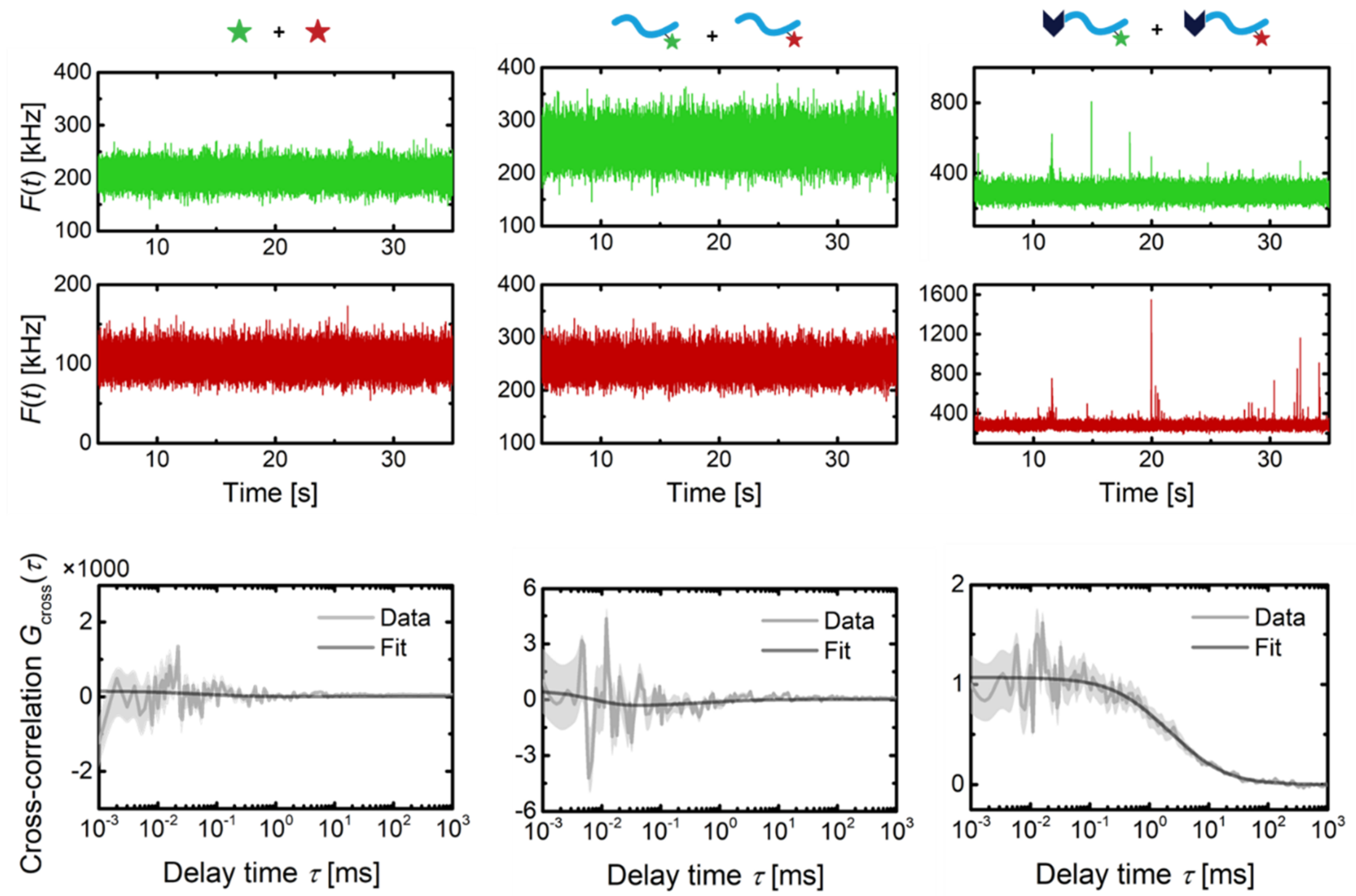

Figure S19. Representative fluorescence fluctuation time traces ($F(t)$, top two rows) and cross-correlations (bottom row) of a mixture of Atto425 amine and sulfo-Cy5 amine (left), a 50:50 mixture of Atto425-TTC-0-p(DMAc) and sulfo-Cy5-TTC-0-p(DMAc) (middle), and a 50:50 mixture of Atto425-TTC-U_2-p(DMAc) and sulfo-Cy5-TTC-U_2-p(DMAc) (right). All Atto- and sulfo-Cy5-labeled species are shown in green and red, respectively. The experimental cross-correlation curves are shown in light grey together with the shaded area that indicates the experimental error. Fits to the correlation data are shown in dark grey.

S7) Additional AFM images

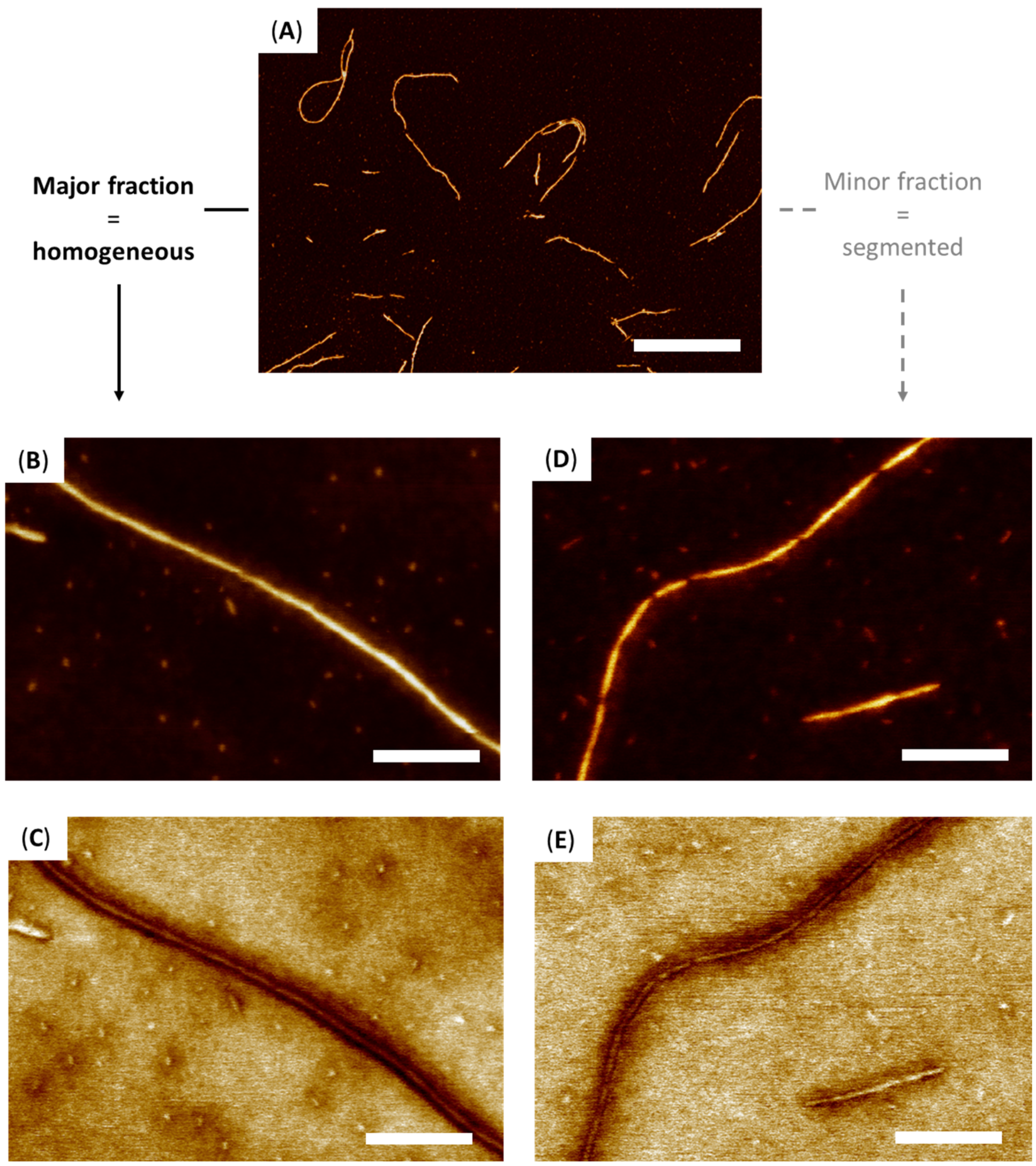

Figure S20. Atomic force microscopy (AFM) characterization of filomicelles (FMs) obtained using solely sticker-containing macroCTAs (TTC-U2-p(DMAc), (**P1**). (**A**) Representative AFM height image with the *z*-axis ranging from 0 to 8 nm. Scale bar = 1000 nm. (**B**, **D**) Height image (*z*-axis: 0 – 8 nm) and (**C**, **E**) corresponding Young's modulus map, where lighter colors indicate stiffer features for an individual homogeneous and segmented FM, respectively. Scale bars = 100 nm. Please note that for **P1** only a small fraction of segmented FMs is observed, while the vast majority of FMs are homogeneous along their contour length. The presence of segmented FMs is attributed to imperfect chain-end fidelity during block copolymer growth, leading to a minor population of non-sticker-containing polymers whose incorporation into the FMs results in mechanically weaker connecting points.

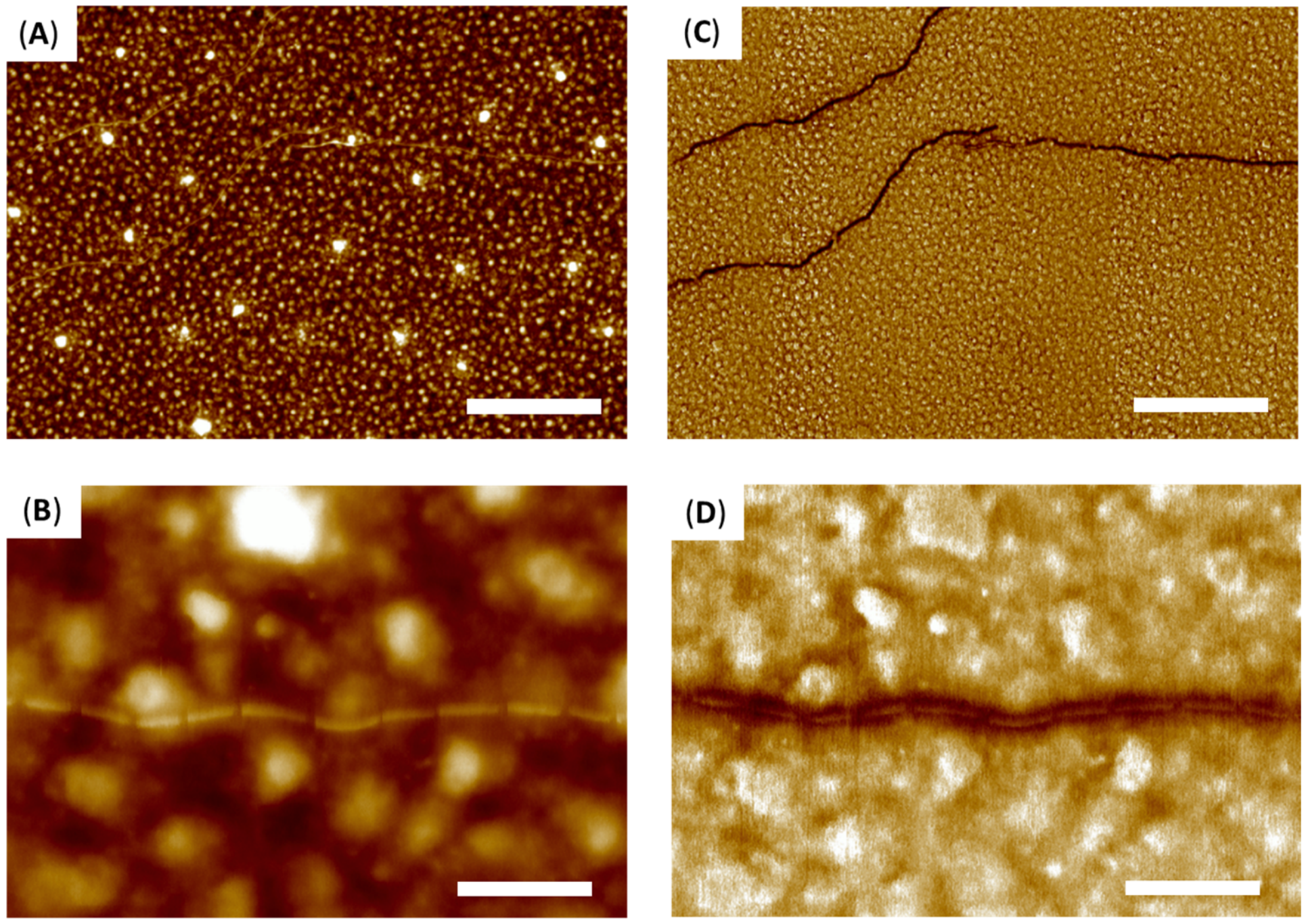

Figure S21. Atomic force microscopy (AFM) characterization of filomicelles (FMs) obtained using a TTC-U_2-p(DMAc):TTC-0-p(DMAc) ratio of 90:10 (**P5**). (**A**) Representative AFM height image with the *z*-axis ranging from 0 to 25 nm and (**C**) corresponding Young's modulus map. Scale bars = 1000 nm. Please note that the presence of large concentrations of spherical particles is in agreement with the results from FCS (**Figure 6** in **Main Text**). (**B**) Higher magnification height image with the *z*-axis ranging 0 to 8 nm and (**D**) corresponding Young's modulus map. Scale bars = 100 nm. For the Young's moduli maps lighter colors indicate stiffer features.

S8) Isolation of FMs via centrifugation

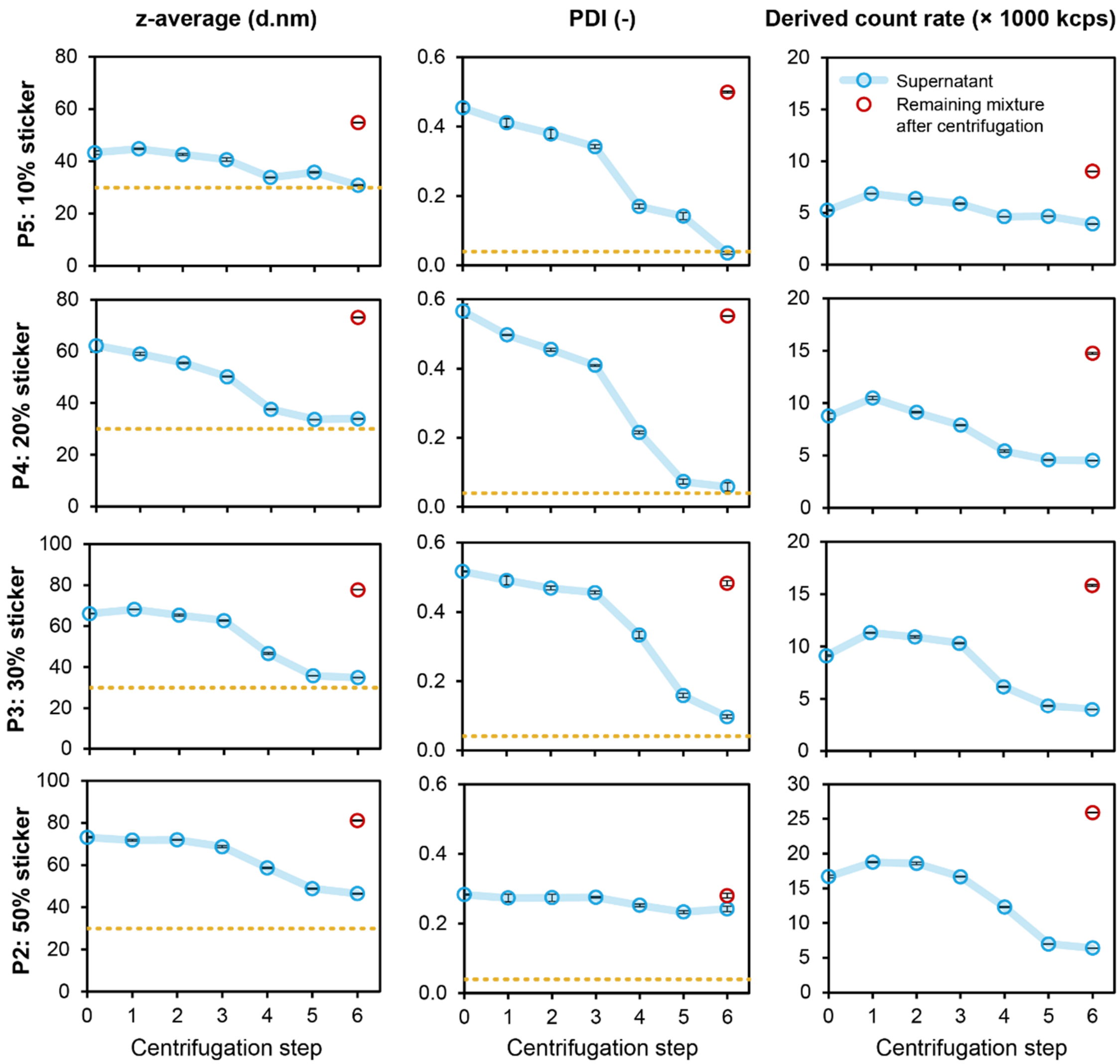

Figure S22. Enrichment of long worms of **P5** (1 mg/mL in water) via centrifugation (stepwise increase of the relative centrifugal force from 500*g* to 20000*g*). DLS results (*z*-average (d.nm) and PDI (-)) of the supernatant (blue circles) after each centrifugation step, as well as of the remaining mixture at the end (red circles).

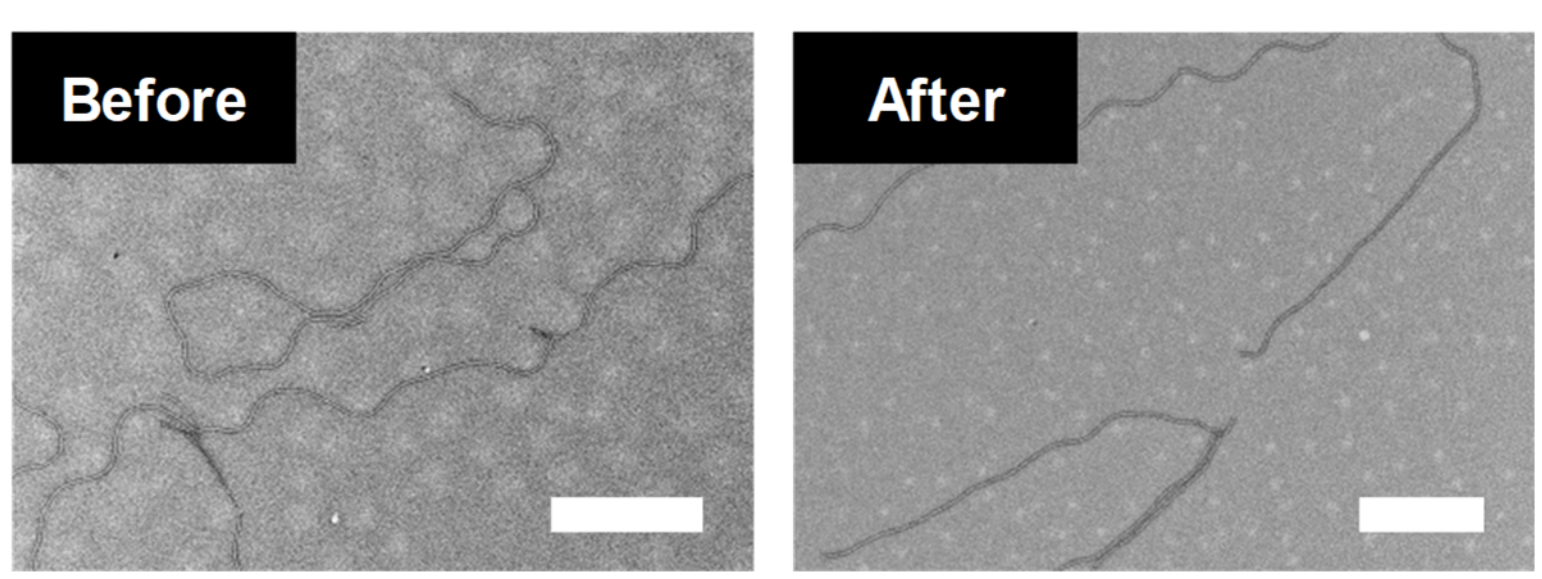

Figure S23. Dry-state TEM images of **P5** before and after the centrifugation protocol. Scale bar = 1 µm.

S9) Shortening via sonication and storage stability

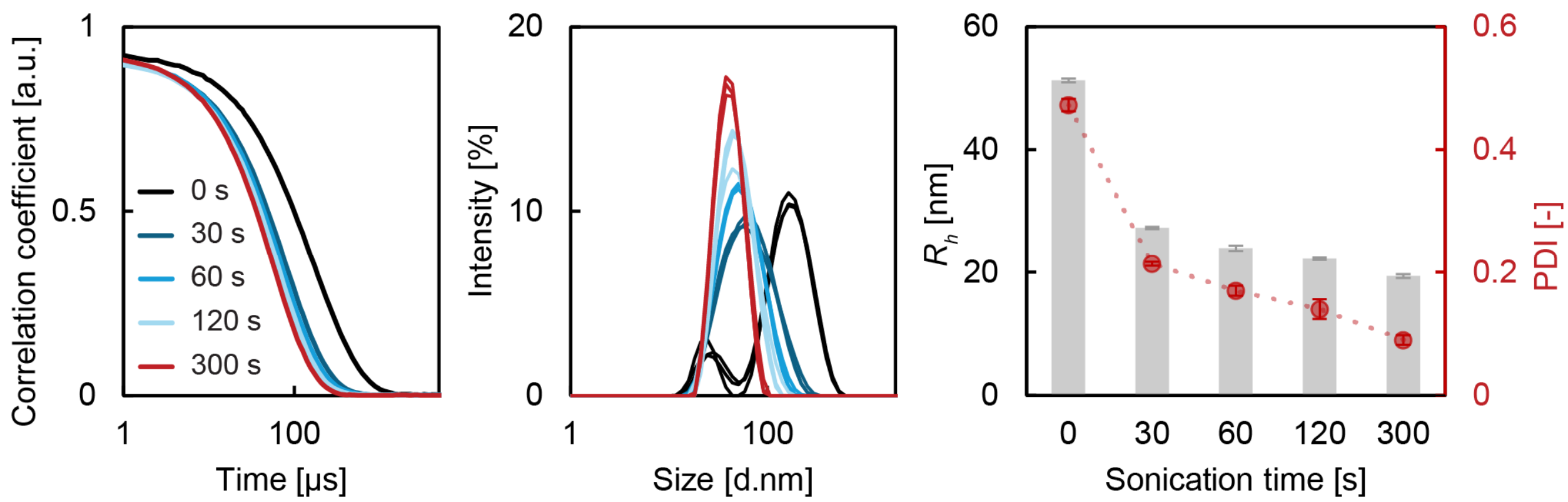

Figure S24. Left: Correlation curves obtained with dynamic light scattering (DLS) for aliquots taken during shortening of the filomicelles (FMs) using sonication. A monotonous decrease in the characteristic delay time is indicative for FM scission. **Middle**: Size distribution by intensity calculated from the correlation function shown on the left. **Right**: hydrodynamic radius of a sphere equivalent R_h (nm, grey bars) and polydispersity (PDI, red circles) after different sonication times (measurements in triplicate, reported error is ± standard deviation).

Table S5. Results from TEM image analysis of sonicated samples with 100% sticker (equivalent to **P1**) on day 0 and after storage for 6 weeks at 4 °C, and at 37 °C with gentle agitation on a roller, everything at 1 mg/mL solid content. The error of L_n is reported as ± standard deviation σ.

**Sample overview**	**Day 0**			**After 6 weeks at 4 °C**			**After 6 weeks at 37 °C with agitation**		
**Sonication duration (s)**	***N*** **(-)**	**$L_n \pm \sigma$ (L_w) (nm)**	**L_w/L_n (-)**	***N*** **(-)**	**$L_n \pm \sigma$ (L_w) (nm)**	**L_w/L_n (-)**	***N*** **(-)**	**$L_n \pm \sigma$ (L_w) (nm)**	**L_w/L_n (-)**
**0**	267	496 ± 554 (1113)	2.2	364	650 ± 682 (1364)	2.1	372	540 ± 654 (1331)	2.5
**30**	140	160 ± 193 (392)	2.5	373	119 ± 115 (230)	1.9	378	147 ± 191 (394)	2.7
**60**	419	118 ± 110 (220)	1.9	395	114 ± 101 (203)	2.0	441	105 ± 111 (223)	2.1
**120**	297	73 ± 32 (87)	1.2	260	79 ± 41 (100)	1.3	449	66 ± 50 (104)	1.6
**300**	345	58 ± 24 (68)	1.2	309	59 ± 39 (85)	1.4	408	75 ± 38 (94)	1.3

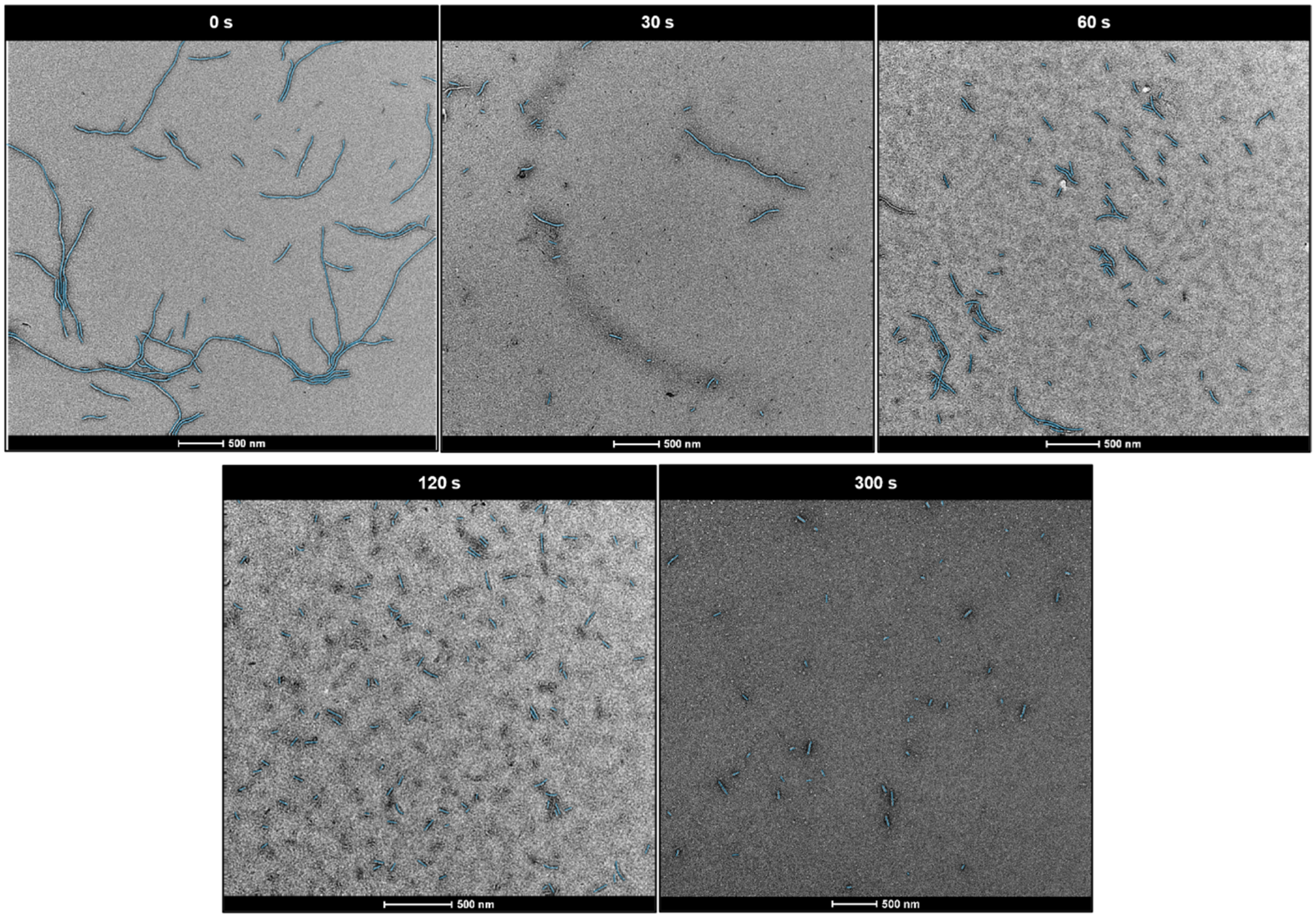

Figure S25. TEM micrographs of **P1** (100% sticker), sonicated for 0, 30, 60, 120, and 300 s, complementary to zoomed images shown in **Figure 8B** in the **Main Text**.

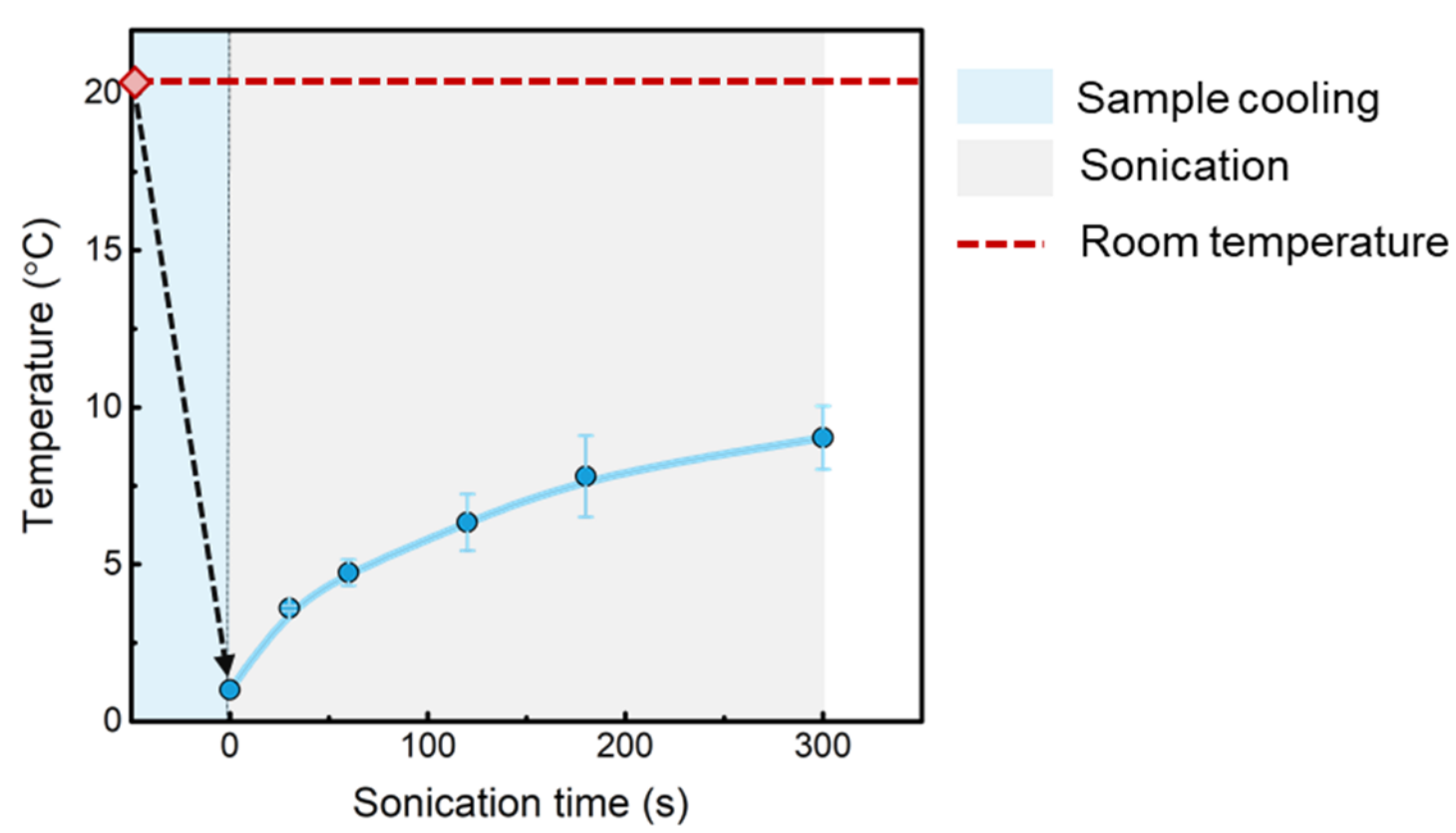

Figure S26. Temperature of an aqueous sample (5 mL) during sonication as described in **Section S1.5** of the **Materials & Methods**. The sample is prepared at room temperature (red) and subsequently placed in an ice bath. This reduces the temperature to 1 – 2 °C (blue shaded area). During sonication (grey shaded area), the temperature gradually increases to a maximum of approximately 9 °C after 300 s. The temperature was measured with a small thermo-couple submerged in the sample during three individual runs. Variations in temperature within these runs are depicted by the error bars. Considering that the samples do not heat up excessively, thermal degradation or disintegration of the filomicelles was ruled out.

REFERENCES

1 G. Mellot, P. Beaunier, J. Guigner, L. Bouteiller, J. Rieger and F. Stoffelbach, *Macromol. Rapid Commun.*, 2019, **40**, 1800315.

2 J. R. Finnegan, X. He, S. T. G. Street, J. D. Garcia-Hernandez, D. W. Hayward, R. L. Harniman, R. M. Richardson, G. R. Whittell and I. Manners, *J. Am. Chem. Soc.*, 2018, **140**, 17127–17140.

3 K. Manalastas-Cantos, P. V. Konarev, N. R. Hajizadeh, A. G. Kikhney, M. V. Petoukhov, D. S. Molodenskiy, A. Panjkovich, H. D. T. Mertens, A. Gruzinov, C. Borges, C. M. Jeffries, D. I. Sverguna and D. Frankea, *J. Appl. Crystallogr.*, 2021, **54**, 343–355.

4 W. R. Chen, P. D. Butler and L. J. Magid, *Langmuir*, 2006, **22**, 6539–6548.

5 J. S. Pedersen and P. Schurtenberger, *Macromolecules*, 1996, **29**, 7602–7612.

6 G. Lamour, J. B. Kirkegaard, H. Li, T. P. J. Knowles and J. Gsponer, *Source Code Biol. Med.*, 2014, **9**, 1–6.

7 M. Rubinstein and R. H. Colby, *Polymer Physics*, Oxford University Press, New York, NY, 1st ed., 2003.

8 Y. Miyaki, Y. Einaga, H. Fujita and M. Fukuda, *Macromolecules*, 1980, **13**, 588–592.

9 T. G. Fox and P. J. Flory, *Journal of Polymer Science*, 1954, **14**, 315–319.

10 T. G. Fox and P. J. Flory, *J. Appl. Phys.*, 1950, **21**, 581–591.

11 L. Trossarelli and M. Meirone, *Journal of Polymer Science*, 1962, **57**, 445–452.
